# Toward a Better Understanding of Bioassays for the Development of Biopharmaceuticals by Exploring the Structure-Antibody-Dependent Cellular Cytotoxicity Relationship in Human Primary Cells

**DOI:** 10.3389/fimmu.2020.552596

**Published:** 2020-10-29

**Authors:** Sébastien Wieckowski, Cécile Avenal, Arturo V. Orjalo, Daniel Gygax, Florian Cymer

**Affiliations:** ^1^ School of Life Sciences, Institute for Chemistry and Bioanalytics, University of Applied Life Sciences and Arts Northwestern Switzerland (FHNW), Muttenz, Switzerland; ^2^Department PTDE-A, F. Hoffmann-La Roche Ltd., Basel, Switzerland; ^3^Biological Technologies, Genentech, Inc., South San Francisco, CA, United States

**Keywords:** breast cancer, FcγRIIIA, flow cytometry, natural killer cells, glycosylation, trastuzumab, antibody-dependent cell-mediated cytotoxicity

## Abstract

Pharmaceutical manufacturing relies on rigorous methods of quality control of drugs and in particular of the physico-chemical and functional characterizations of monoclonal antibodies. To that end, robust bioassays are very often limited to reporter gene assays and the use of immortalized cell lines that are supposed to mimic immune cells such as natural killer (NK) cells to the detriment of primary materials, which are appreciated for their biological validity but are also difficult to exploit due to the great diversity between individuals. Here, we characterized the phenotype of the peripheral blood circulating cytotoxic cells of 30 healthy donors, in particular the repertoire of cytotoxic markers, using flow cytometry. In parallel, we characterized the antibody-dependent cellular cytotoxicity (ADCC) effector functions of these primary cells by measuring their cytolytic activity against a cancer cell-line expressing HER2 in the presence of trastuzumab and with regards to FCGR3A genotype. We could not establish a correlation or grouping of individuals using the data generated from whole peripheral blood mononuclear cells, however the isolation of the CD56-positive population, which is composed not only of NK cells but also of natural killer T (NKT) and γδ-T cells, as well as subsets of activated cytotoxic T cells, monocytes and dendritic cells, made it possible to standardize the parameters of the ADCC and enhance the overall functional avidity without however eliminating the inter-individual diversity. Finally, the use of primary CD56^+^ cells in ADCC experiments comparing glycoengineered variants of trastuzumab was conclusive to test the limits of this type of *ex vivo* system. Although the effector functions of CD56^+^ cells reflected to some extent the *in vitro* receptor binding properties and cytolytic activity data using NK92 cells, as previously published, reaching a functional avidity plateau could limit their use in a quality control framework.

## Introduction

The development of therapeutic and preventive antibodies requires a high standard quality control of structural and functional characterization as well as defining specifications that are in line with regulatory guidance established by regulatory authorities and the International Council for Harmonization (ICH) (1, 2). Not only the proper characterization of critical quality attributes and assessment of immunogenicity is important during the development process of biopharmaceuticals (3), but characterizing the biological functions at the cellular level is essential to guide manufacturing of the antibody-based drug (4). However, in this good manufacturing practice (GMP) based context of quality control, the requirements for precision, accuracy and statistical power often necessitates neglecting the qualitative and quantitative evaluation of underlying and highly complex biological processes of the drug in parallel. Furthermore, as knowledge progresses during the often-long development timelines of biopharmaceuticals, previous assumptions made for analytical methods used in quality control have to be continuously challenged.

One of the most representative example is the development of therapeutic antibodies such as antibodies for the immunotherapy of cancer, and in particular antibodies capable of anti-tumor activities such as direct cytotoxicity and interference with cell signaling, as well as mechanisms mediated through Fc receptors (FcRs), in particular FcγRs, of competent cytotoxic and phagocytic innate immune cells such as antibody-dependent cellular cytotoxicity (ADCC) (5), antibody-dependent cellular phagocytosis (ADCP) and immune complex uptake (6). Based on the antibody format and structure, e.g., bi-specific antibodies, bi-specific T-cell engagers (BiTEs) or Triomab, additional and sometimes complementary anti-cancer strategies can be undertaken via the engagement of the adaptive arm of the immune system. Finally, fine tuning and feature engineering such as modification of N-linked glycosylation (7) affect the binding affinity of the Fc domain to its cognate receptors (8), affect the ADCC activity *in vitro* and *in vivo* (9–11), ultimately leading to enhanced clinical responses (12, 13).

While ADCC activity is better assessed by using primary cytotoxic cells, such assessments are often substituted by more robust bioassays using cell lines such as NK92 (14) or engineered Jurkat cells (15). The Jurkat cells are in fact used as a cellular system reporting the very first step of ADCC, i.e., the binding of a cognate antibody-tumor complex to an exogenous FcγRIIIa or CD16a, the induction of a signaling cascade from the immunoreceptor tyrosine-based activation motif (ITAM) (16) and the phosphorylation of NFAT2 and calcium flux (17), ultimately resulting in luminescence. In addition to the complete lack of cytolytic activity, these cells express CD16a at a very low level, as compared to primary natural killer (NK) cells and NK92 (18). The human NK92 cell line has a malignant non-Hodgkin’s lymphoma origin and its growth depends on exogenous IL-2. Therefore, it can be considered a rather artificial cell line with CD16a expression. The set of NK92 signaling pathways engaged toward the exocytosis of lytic granules necessary for the cytotoxic activity (19, 20) and ADCC properties are reasonably comparable to that of primary NK cells (21), although the biological complexity and plasticity of the expression of the numerous surface markers are not entirely representative of the biological reality.

Indeed NK cells are much more diverse and varied than what was assumed a few years (22), before the advent of new high throughput analysis technologies such as mass cytometry (CyTOF) (23) and sequencing at the single cell (24). It is now known, that NK cells do not only express CD56 and CD16 at varying levels (25, 26), but also display a wide array of activating and inhibiting receptors such as natural cytotoxicity receptors (NCRs) (27), killer-cell immunoglobulin-like receptors (KIRs) (28), and killer lectin like receptors (KLRs) (29), as well as cytokine and chemokine receptors and adhesion molecules. All these receptors regulate the cell signaling downstream of the FcR and the cytotoxic effector functions, and depicting the NK repertoire (30). It is also important to take into account the single-nucleotide polymorphism (SNP) of the *Fcgr3a* gene which has been widely described in the literature as so-called “high affinity” and “low affinity” FcγRIIIa haplotypes, among other polymorphisms (31, 32) and copy number variations (33), and has been shown to be important for the clinical responses during treatment with trastuzumab (34–36), although still debated (37).

NK cells can then be grouped into a broad spectrum of subsets constituting a real continuum in the development of NK cell lineage rather than distinct end-stage subgroups (38–40). Equally remarkable is the diversity between healthy individuals (41) or at different ages (42), but also between different anatomic sites in the same individual or in a dysregulated context due to a disease such as in the tumor context (43–45) or a viral infection (46). For example, an increased proportion of more immature and noncytotoxic NK cell subsets was observed in the peripheral blood of patients with breast cancer, accounting for the low cytotoxic functions measured in these patients (47). Not only NK but also natural killer T (NKT) cells (48, 49), γδ-T cells (50, 51), subsets of T lymphocytes (52), dendritic cells (DCs) (53) as well as monocytes and macrophages (54–56), can mediate ADCC. All of these effector cells are fine-tuned by a broader immune contexture composed of multiple helper and regulatory cells such as tissue-resident lymphocytes (T_RM_) (57).

With an increasing understanding of the potential clinical implications of these discoveries, and more commonly applied structural modifications of therapeutic antibodies (11), it seemed urgent to us to clarify whether the potency assays utilized during the last stages of pharmaceutical drug development sufficiently reflect the underlying biological reality. The aim of this study was therefore to address the cell diversity of circulating primary effector cytotoxic cells in thirty (30) healthy individuals, in the context of ADCC induced with trastuzumab using a HER2/neu-expressing breast cancer cell line (58–60), and to determine the key variables of ADCC and their relation with the structural and functional status of the cytotoxic cells. We furthermore evaluated the effects of structural modifications of trastuzumab on ADCC and assessed the reasonability of quality control assay simplifications, discussing the limits of bioassays utilizing primary cells within the framework of the development of therapeutic antibodies with ADCC activity.

## Results

### The Diversity in the Composition of the Immune System Cells in the Blood of Healthy Donors

**Figure 1:**
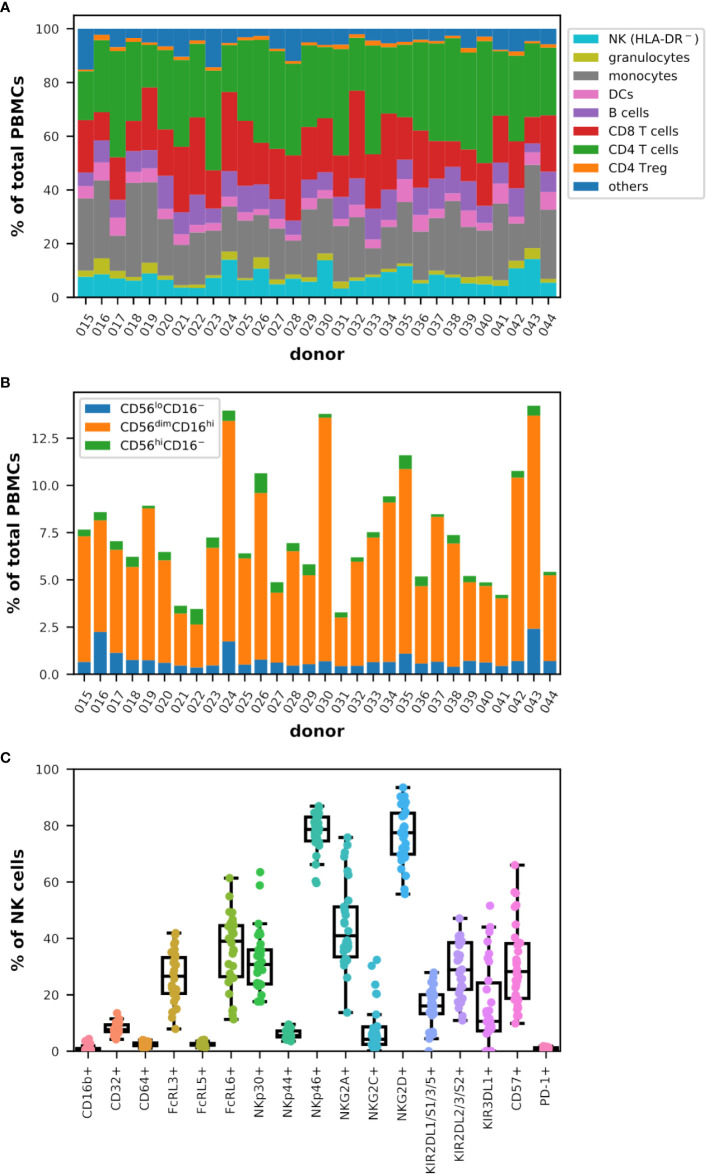
Diversity in the representation of the main immune populations and subgroups of cytotoxic cells in the blood of healthy donors analyzed by flow cytometry (n = 30). **(A)** Cumulative bar graph representing the frequency of the populations indicated in the legend among total live PBMCs for each donor. Note that HLA-DR^−^ NK cells were measured. **(B)** Cumulative bar graph plotting the frequency of the three major subsets of NK cells (CD56^+^lineage^−^) as indicated in the legend among total live PBMCs for each donor. **(C)** Box plots overlaid with strip plots summarizing the frequency of NK cells (CD56^+^lineage^−^) expressing the indicated marker.

The main goal of this study was to analyze the potential relationships between the phenotype of immune cells isolated from the blood of healthy donors, and ADCC activities of cytotoxic cells toward HER2-expressing target cells in the presence of trastuzumab. To this end, we have first designed and optimized three panels of antibodies for flow cytometry analysis of the various immune cell populations and subsets of cytotoxic cells. A first panel was designed for the analysis of FcR and Fc receptor-like (FcRL) receptors, a second panel for the analysis of important markers of cytotoxicity, in particular the NCR, KIR and KLR markers, and finally a third panel based on OMIP-41 (61) for the most comprehensive possible immunophenotyping of the major lymphocyte and myeloid subsets (**Table S1** and **Figure S1**). It should be noted that the gating strategy using this latter panel excludes HLA-DR positive cells from the NK cell population, which may obscure the presence of adaptive NK cells (62). The frequency of NK cells measured using this third panel and shown in **Figure 1A** may then differ from that measured using the other panels which allow detection of the total NK cell population which is discussed in the rest of this study. Furthermore, NKT cells comprise a unique subset of CD1d-restricted T cells with characteristics of both NK and T cells (63) and whose rigorous identification requires CD1d-α-GalCer tetramers, which was not performed in this study. Our sampling of 30 donors is fairly balanced, with 12 women and 18 men, being between 20.0 and 74.0 years old with an average age of 43.1 ± 15.1 all sexes combined as summarized in **Table 1**.

**Table 1:** Donor summary demographics.

Sex	Age (mean ± SD)	N (%)
Female	35.4 ± 11.7	12 (40)
Male	48.2 ± 15.2	18 (60)

The distribution of CD4^+^ helper (T_H_) and CD8^+^ T cells, as well as regulatory T cells (T_reg_), B lymphocytes, dendritic cells (DCs), monocytes, granulocytes, and NK cells (HLA-DR^−^), was diverse and varied and specific to each donor as summarized in **Figure 1A**, and it was therefore impossible to classify donors in a reasonable manner according to these data. Other markers have been included in this analysis (raw data are publicly available) but are not discussed in more detail as they did not allow to improve classification (data not shown). The frequency values among the viable peripheral blood mononuclear cells (PBMCs) ranged between 3.3% and 14.3% for NK cells (HLA-DR^−^), 0.7% and 5.9% for granulocytes, 9.7% and 35.0% for monocytes, 2.2% and 8.6% for DCs, 3.3% and 12.3% for B cells, 9.5% and 32.6% for CD8^+^ T cells, 15.9% and 45.4% for T_H_, 0.6% and 2.0% for T_reg_, and 2.0% and 15.2% for other immune cells, which include NKT and γδ-T cells and some basophils (**Figure S1D**). When investigating more closely the NK cells and in particular the three main subsets defined by the relative expression of CD56 and FcγRIII (CD16), the diversity was no less complex among the donors analyzed as shown in **Figure 1B**. However, the range of frequency values for each subtype corresponded to the normal rates observed by others in the peripheral blood of healthy donors (25, 64, 65) with between 0.4% and 2.4% of CD56^lo^CD16^−^ cells expressing low levels of CD56 and no CD16, 2.3% and 12.9% of CD56^dim^CD16^hi^ cells expressing intermediate levels of CD56 and high levels of CD16 and which remained the majority in the NK cell population, and 0.1% and 1.0% of CD56^hi^CD16^−^ cells expressing high levels of CD56 and no CD16 among the viable PBMCs.

Finally, the analysis of the expression of cytotoxic markers on NK cells confirmed that each donor tends to be unique as shown in **Figure 1C**. Although the near absence of expression of CD16b (1.0% ± 1.1), PD-1 (1.0% ± 0.4), CD64 (FcγRI; 2.4% ± 0.7) and FcRL5 (2.5% ± 0.7) on NK cells in all the donors analyzed, and the consistent low frequency of NKp44^+^ (6.1% ± 1.6) and CD32^+^ (FcγRII; 7.9% ± 2.1), these frequencies extended significantly in different donors for markers such as NKp46 (from 59.6% to 86.9% of NK cells), KIR2DL1/S1/3/5 (0.0%–27.9%), NKG2C (0.2%–32.4%), FcRL3 (7.9%–41.9%), KIR2DL2/3/S2 (10.9%–47.1%), NKG2D (55.7%–93.5%), NKp30 (17.6%–63.5%), and more dramatically FcRL6 (11.3%–61.4%), KIR3DL1 (0.0%–51.6%), CD57 (9.8%–66.0%), and NKG2A (13.7%–75.8%). Similar values have been obtained by others (63). Interestingly, KIR3DL1 was nearly absent from NK cells in 6 out of 30 donors while NKG2C was expressed in more than 8% of the NK cells in 9 donors (**Figure S2**).

### Correlation Between the Expression of Cytotoxic Markers, the FCGR3A Haplotype and ADCC Activity in PBMCs

We then measured the cytolytic activity in an ADCC system in which BT474 target cells, expressing HER2 at a very high level (66) (data not shown), are co-cultured with PBMCs from the different healthy donors in the presence of increasing concentrations of trastuzumab. We included an additional layer of information by taking into consideration the FCGR3A-158 haplotype of each donor, which was obtained by genotyping using Droplet Digital™ PCR (**Figure S3**). Our donor base was thus composed of 13 donors homozygous for the FCGR3A-158 F allotype, 14 heterozygous donors FCGR3A-158 F/V and 3 donors homozygous for the FCGR3A-158 V allotype (**Table S2** and **Figure S3D**), which corresponded to the regular distribution of this SNP as observed by others (32, 33, 35).

**Figure 2:**
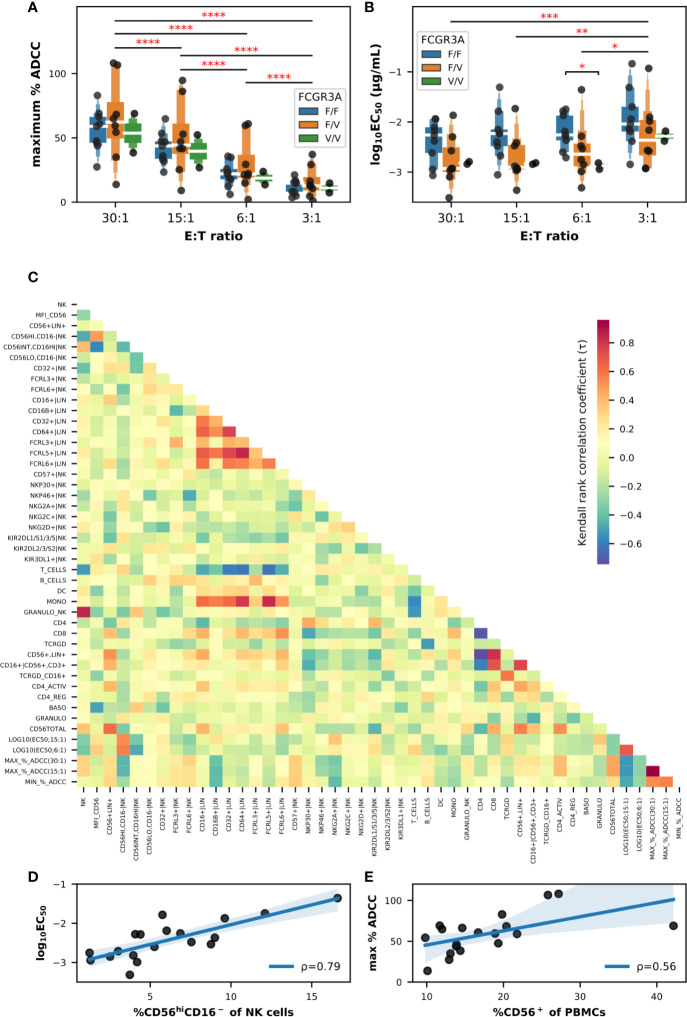
Impact of the FCGR3A-158 haplotype on the ADCC effector functions of PBMCs against BT474 target cells in the presence of trastuzumab, and correlation analyses between flow cytometry and ADCC data (n = 19). Letter-Value box plots overlaid with strip plots summarizing **(A)** the maximum percent of specific lysis, and **(B)** the EC_50_ values (log_10_-transformed), obtained using total primary PBMCs as effector cells from donors with the FCGR3A-158 haplotype indicated in the legend, and the E:T ratios indicated on the x-axis. **(C)** Heatmap summarizing the association (Kendall rank coefficients) between the different parameters analyzed. Note that the EC_50_ values were selected using an E:T ratios of 6:1 and 15:1, while the maximum and minimum percentages of ADCC activity were selected using an E:T ratios of 15:1 and 30:1. Linear correlations **(D)** between the EC_50_ value (log_10_-transformed; E:T = 6:1) and the frequency of NK cells with the CD56^hi^CD16^−^ phenotype, and **(E)** between the maximum percent of specific lysis (E:T = 30:1) and the frequency of total CD56^+^ cells among PBMCs. The value of the Pearson coefficient (ρ) is indicated for each linear regression result, with P values of 0.001 in **(D)** and 0.0127 in **(E)**. In **(A**, **B)**, only statistically significant results from T-tests corrected for multiple-comparisons after mixed-design ANOVA are shown and are represented by red asterisks. ****P ≤ 0.0001, ***P ≤ 0.001, **P ≤ 0.01, *P ≤ 0.05.

Firstly, we evaluated the influence of the FCGR3A-158 haplotype as well as the effector-to-target ratio (E:T) on a sample of PBMCs from 19 donors. The maximum percentage of specific lysis or ADCC (**Figure 2A**) as well as the EC_50_ values (**Figure 2B**) were extracted from the sigmoid curves defining a four parameter logistic (4PL) regression and fitting the titration data points for each donor and E:T tested. We noticed a certain disparity in the ADCC capacity of PBMCs between the donors, for some the specific cytolytic activity was high starting from the lowest E:T ratio, e.g., in donors 024 and 030, for others this activity remained weak even at an E:T of 30:1, e.g., in donors 018 and 021 (**Figure S4A**). This disparity among the PBMCs from the different donors translated to a heterogeneous distribution of the maximum percentages of specific lysis in the box plots shown in **Figure 2A**, these values ranging from 9.1% to 94.7% all FCGR3A-158 haplotypes combined and an E:T ratio of 15:1. Although neither the effect of the FCGR3A-158 haplotype (F(2,16) = 0.375; P > 0.05) nor of the interaction between the E:T ratio and the FCGR3A-158 haplotype (F(6,48) = 0.199; P > 0.05) was significant, that of the E:T was indeed (F(3,48) = 139.7; P < 0.0001) according to mixed-design ANOVA. Thus the average maximum percentages of specific lysis correlated with the E:T ratio, with mean values reaching 13.4% ± 8.8, 24.4% ± 15.2, 45.2% ± 20.6 and 59.0% ± 23.8 for E:T ratios of 3:1, 6:1, 15:1, and 30:1, respectively, independently of the FCGR3A-158 haplotype. Moreover the lower asymptote of the fitting sigmoid curve, i.e., the baseline natural cytolytic activity, was very low especially for E:T ratios below 15:1 with values for specific lysis not exceeding 8.5%, and moderate only in donors 025, 030, and 034 for the highest E:T ratio (**Figure S4B**).

The results of the analysis of EC_50_ values was less conclusive (**Figure 2B**), and although the effect of the E:T ratio was significant according to mixed-design ANOVA (F(3,48) = 12.21; P < 0.0001), the effect of the FCGR3A-158 haplotype (F(2,16) = 2.05; P > 0.05) and the interaction between the E:T ratio and the FCGR3A-158 haplotype (F(6,48) = 0.327; P > 0.05) were not. A trend emerged for the effect of the FCGR3A-158 haplotype which appeared to be significant between the groups FCGR3A-158 F/F and FCGR3A-158 V/V using *post hoc* unpaired T-tests (corrected P = 0.017), more precisely at the E:T ratio of 6:1 (corrected P = 0.010). Thus the EC_50_ values decreased with an increasing E:T ratio, with mean EC_50_ values of 7.91, 3.73, 3.09, and 2.56 ng/ml for E:T ratios of 3:1, 6:1, 15:1, and 30:1, respectively, and tended to decrease, i.e., the affinity increased, in homozygous FCGR3A-158 V/V compared to homozygous FCGR3A-158 F/F donors, although the number of donors in the first group was too low for consolidation. The functional avidity was much higher with the PBMCs than with NK92 cells transfected with the low (NK92-158F) and high affinity (NK92-158V) CD16 receptor (14), as used as internal controls with an E:T ratio of 5:1, with EC_50_ values of 255.5 and 33.2 ng/ml, respectively (data not shown), in accordance with data published by others (18).

We then analyzed the potential relationships between flow cytometry and ADCC data in the PBMCs of the same set of donors. We had initially used all the data available (raw data are publicly available) and we limited the shown data here to the most significant, in order not to confuse the message. The results of non-parametric pairwise correlation measurements using the Kendall Tau test are summarized in the heatmap shown in **Figure 2C**. The τ coefficients were generally close to zero, indicating that there was little or no correlation between the parameters analyzed, and most of the relationships found to be significant were between parameters independent of the ADCC such as the inverse correlations between the frequency of total T cells and those of monocytes, between the frequency of B cells and those of γδ-T cells, or between the frequency of T cells and those of NK cells. Furthermore, positive correlations were observed between the frequency of CD8^+^ T cells and the frequency of CD56^+^lineage^+^ cells, and between the frequency of FCGR- and FcRL-expressing cells in the lineage^+^ population and the frequency of monocytes in the PBMCs. Note that the trend in correlation coefficients was similar when comparing the functional avidity data at E:T ratios of 6:1 and 15:1, as well as the maximum lysis values at E:T ratios of 15:1 and 30:1 (**Figure 2C**).

Only two linear correlations involving the cytolytic parameters appeared stronger than others and concerned the relationship (a) between the functional avidity represented by the common logarithm of the EC_50_ value, in particular at the E:T ratio of 6:1, and the frequency of CD56^hi^CD16^−^ NK cells (**Figure 2D**), and (b) between the maximum specific lysis values, e.g., at the E:T ratio of 30:1, and the overall frequency of total CD56^+^ cells in PBMCs (**Figure 2E**), with Pearson coefficients of 0.79 (P = 0.0001) and 0.56 (P = 0.0127), respectively. Attempts at dimensional reduction using principal component analysis (PCA) and different combinations of flow cytometry datasets obtained on total PBMCs did not result in any obvious cluster (**Figure S5**). Therefore, and based on the importance of CD56, a phenotypic marker expressed not only on NK cells but also on NKT and γδ-T cells as well as on certain subsets of T lymphocytes, dendritic and monocytic cells (26) with immunostimulatory effector functions, we concentrated our efforts on the analysis of CD56^+^ cells and used isolated CD56^+^ cells in ADCC assays, while increasing the number of individuals up to thirty.

### Structure-Effector Function Relationship Study Using Isolated CD56^+^ Cells

**Figure 3:**
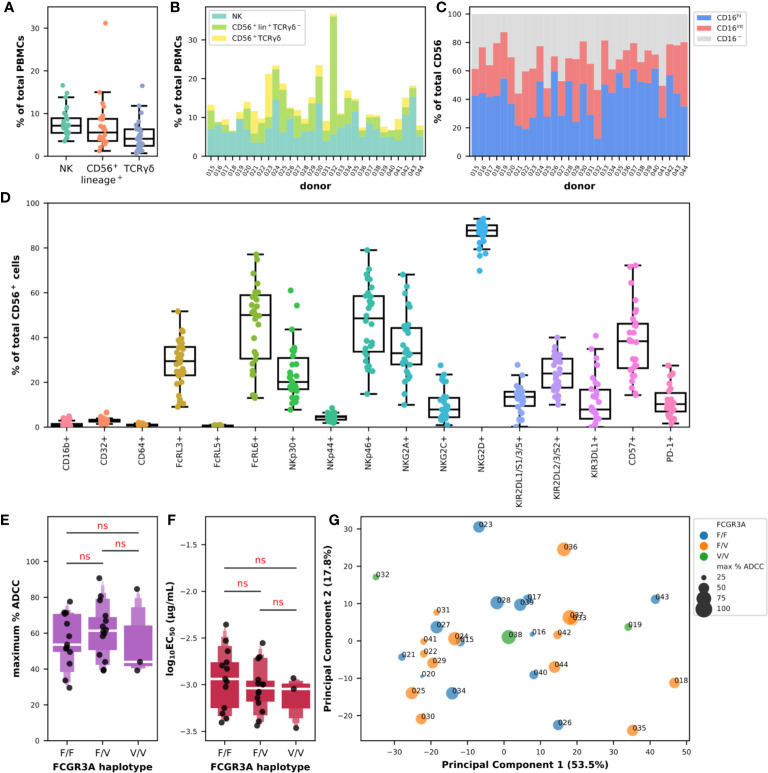
Diversity of cytotoxic markers on total CD56-positive cells and relationships with their ADCC effector functions against BT474 target cells in the presence of trastuzumab. **(A)** Box plots overlaid with strip plots summarizing the frequency of NK (CD56^+^lineage^−^), CD56^+^lineage^+^ that include NKT, γδ T cells (lineage^+^TCRγδ-1^+^), and other effector cells among total PBMCs (n = 30). **(B)** Cumulative bar graph showing the frequency of NK (CD56^+^lineage^−^TCRγδ-1^−^), CD56^+^lineage^+^TCRγδ-1^−^, and γδ-T (CD56^+^lineage^+^TCRγδ-1^+^) cells among CD56^+^ cells in each donor. **(C)** Cumulative bar graph showing the frequency of cells expressing high (CD16^hi^) and intermediate levels (CD16^int^) of CD16, or no CD16 (CD16^−^) among CD56^+^ cells in each donor. **(D)** Box plots overlaid with strip plots summarizing the frequency of CD56^+^ cells expressing the indicated marker (n = 30). **(E)** Letter-Value box plots overlaid with strip plots summarizing the maximum percent of specific lysis, and **(F)** the EC_50_ values (log_10_-transformed), obtained using isolated CD56-positive effector cells from donors with the FCGR3A-158 haplotype indicated on the x-axis, and an E:T ratio of 5:1. **(G)** Multidimensional bubble plot showing the result from a PCA carried out using the flow cytometry data of the expression of the NCR, KIR and KLR markers on the CD56-positive population. Each data point summarizes for each donor its FCGR3A haplotype and the value of the maximum percent of specific lysis encoded with colors and sizes as indicated in the legend. Values in parentheses show the percentage variance explained by each PCA axis. All the data used in this figure were obtained from experiments fully independent of the experiments presented in **Figure 2**. In **(D**, **E)**, the results of pairwise Tukey-HSD T-tests corrected for multiple-comparisons performed after one-way ANOVA are indicated in red. ns: not significant.

We first analyzed the composition of PBMCs in true NK cells, defined as CD56^+^lineage^−^, CD56^+^lineage^+^ cells which include not only NKT cells but also subsets of γδ and activated T lymphocytes, and total (and not necessarily CD56^+^) γδ-T cells defined as lineage^+^ cells expressing γδ-TCR, which respectively represented 7.7% ± 3.2, 7.0% ± 5.6, and 4.7% ± 3.5 of total PBMCs (**Figure 3A**). The diversity in the composition of subpopulations of CD56^+^ cells appeared evident when comparing the proportions of NK, CD56^+^lineage^+^TCRγδ-1^−^, and γδ-T cells in each donor as shown in **Figure 3B**. The cumulative frequency of CD56^+^ cells varied between 6.6% and 36.7% with a mean at 13.5% ± 6.4 which could provide part of the explanation for the differences observed in ADCC activity using total PBMCs between certain donors. NK cells did not always represent the main subpopulation of the CD56-positive fraction, despite an average of 59.3% ± 19.5, 27.9% ± 17.1, and 12.8% ± 12.5 for NK, CD56^+^lineage^+^TCRγδ-1^−^, and γδ-T cells, respectively. Indeed, the proportion of the population we identified as CD56^+^lineage^+^TCRγδ-1^−^ cells could reach up to 82.7% of CD56^+^ cells as in donor 032, and represented more than 40.0% in 5 donors, and γδ-T cells up to 52.5% as in donor 023, and represented more than 25.0% in 4 donors (**Figure 3B**). Analysis of the expression of FcγRIII in the overall CD56^+^ population also indicated some degree of heterogeneity with a mean frequency of cells expressing CD16 of 68.2% ± 11.5 with values falling below 50% in 4 donors (**Figure 3B**). The distribution of cells expressing high and intermediate levels of CD16 also varied substantially, with frequencies ranging between 12.4% and 61.7% for the first and between 9.5% and 41.1% for the latter.

Next, we looked at the expression of the different cytotoxic markers as we did on the sole NK cells in **Figure 1C**, this time by analyzing the entire CD56-positive population (**Figure 3D**). The expression patterns of the different markers were somewhat comparable, with the near absence of expression of CD16b (1.3% ± 1.4), CD64 (1.0% ± 0.4) and FcRL5 (0.6% ± 0.2), and the low frequency of NKp44^+^ (4.4% ± 1.4) and CD32^+^ (3.0% ± 1.0) cells in the CD56-positive population. The rates and the levels of variation were also comparable to the ones observed on NK cells for the markers NKG2C (0.9%–27.6%), KIR2DL1/S1/3/5 (0.2%–27.8%), KIR2DL2/3/S2 (10.0%–40.0%), KIR3DL1 (0.0%–40.8%), FcRL3 (9.0%–51.7%), NKp30 (7.8%–61.0%), CD57 (14.3%–72.2%), NKG2A (9.9%–68.1%), and FcRL6 (13.0%–77.1%). Interestingly, the proportion of CD56^+^ cells expressing PD-1 was much higher than in NK cells with 11.1% ± 7.4 of the cells expressing this exhaustion marker. Moreover, the frequency of cells expressing NKG2D seemed to have stabilized toward higher levels around 86.6% ± 5.4 in CD56^+^ cells, and the values of those expressing NKp46 became on the contrary more heterogeneous than on NK cells with values ranging between 14.8% and 79.0%. It was finally interesting to note the significant higher expression of certain markers in the CD56^+^lineage^+^ population such as CD16b, CD32, CD64, FcRL5, and PD-1, and the drastic reduction in expression of all the NCR/KIR/KLR markers apart from NKG2D (**Figure S6**), making us think of a myeloid signature.

In the rest of this study, we analyzed the cytolytic activity of CD56^+^ obtained after immunomagnetic cell separation from PBMCs and no longer whole PBMCs, in the presence of BT474 tumor target cells and trastuzumab. Interesting points emerged despite the use of a single E:T ratio of 5:1. First, the substantial heterogeneity in the ADCC response observed with PBMCs was far less here, with values for the maximum percentage of ADCC ranging from 29.5% to 90.7% and a mean value of 58.6% ± 15.8 for all FCGR3A-158 haplotypes combined (**Figure 3D** and **Figure S7A**). Second, the effect of the FCGR3A-158 haplotype was not significant (F(2,27) = 0.445; P > 0.05) according to one-way ANOVA, and no trend of significant difference could be observed between the three groups of donors using *post hoc* T-tests, in a way comparable to what has been observed with the total PBMCs (**Figure 3A**). Third, the functional avidity was somehow homogenized with the use of isolated CD56^+^ cells with the values of the common logarithm of EC_50_ in µg/ml ranging from −3.5 to −2.4 and a mean EC_50_ of 0.99 ng/ml all FCGR3A-158 haplotypes combined (**Figure S7B**). Likewise, the effect of the FCGR3A-158 haplotype was not significant (F(2,27) = 0.80; P > 0.05) according to one-way ANOVA, and no trend could be observed either between the three FCGR3A-158 haplotype groups (**Figure 3E**). Lastly, the difference in functional avidity between the PBMCs and the isolated CD56-positive cells was significant whether it was analyzed using paired T-tests using data from the 19 donors tested in both experimental settings (the 95% CI for the difference in the means of the common logarithm of EC_50_ when PBMCs were used at an E:T=15:1 was [0.23, 0.76]), or using unpaired T-tests using all the data points ([0.22, 0.72]), which was valid for all the ratios tested (**Figure S7C**).

We finally tried to capture any relationship between cellular markers among the level of expression of FcγR, FcRL, NCR, KLR, and KIR markers on total CD56^+^ cells and the ADCC parameters obtained with the isolated CD56^+^ cells as effector cells but without success (**Figure S8**). We also tried to use all our data in different combinations in dimensional reduction analyses such as PCA, but we were unable to group the donors in any clusters (**Figure S9**). The archetype of these analyses is shown in **Figure 3G** for which only the data for the expression of the NCR/KIR/KLR markers on the CD56^+^ cells were used. We observed an almost uniform distribution of all the data points along the principal components, and this independently of the FCGR3A-158 haplotype and independently of the maximum ADCC activity. It is possible that the relative homogeneity, compared to PBMCs, in the parameters observed here, with the low although reasonable number of donors as well as the limited number of features tested did not make this quest conclusive on the scale of cellular subpopulations.

### Analysis of the Cytotoxic Marker Repertoire of CD56^+^ Cells at the Single-Cell Level

**Figure 4:**
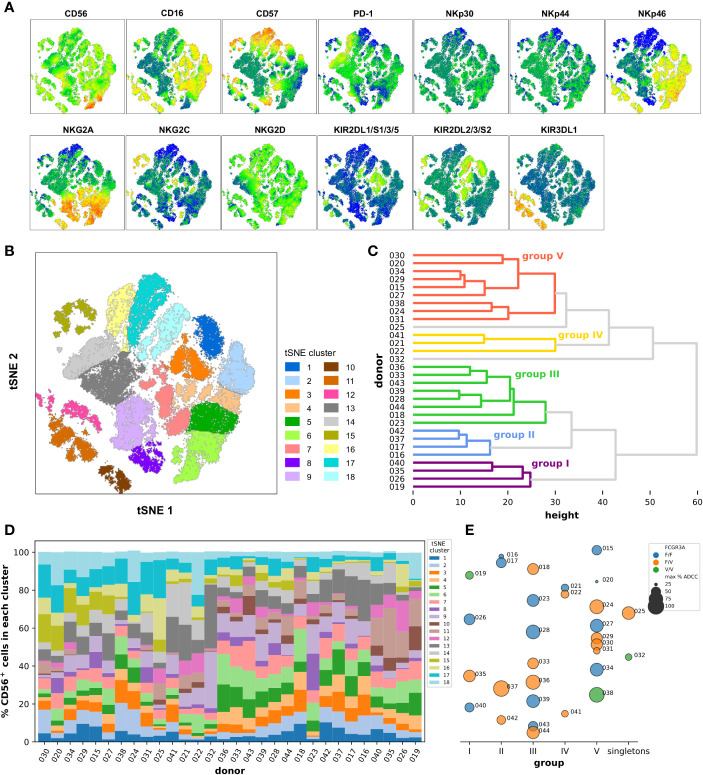
Dimensionality reduction of the flow cytometry data at the single cell level using the cytotoxic marker panel in the CD56^+^ cell population. **(A)** Marker heatmap dot plots obtained after t-SNE and showing the relative expression of the indicated marker in the different phenotypic clusters. **(B)** The phenotypic clusters were delimited manually onto the t-SNE dot plot based on the similarity in the phenotypic profiles of the cells that compose them and coded according to the color indicated in the legend. **(C)** Dendrogram representing the classification of the donors into the groups I to V using hierarchical clustering with a distance cut-off of 32, according to the frequency of their CD56^+^ cells in the different phenotypic clusters defined in **(B)**. **(D)** Frequencies of CD56^+^ cells in the different phenotypic clusters defined in **(B)** in the 30 donors sorted from left to right according to the order obtained from top to bottom after hierarchical clustering as shown in **(C)**. The colors of the phenotypic clusters in **(D)** correspond to those of the clusters from the t-SNE map in **(B)**. **(E)** Underlying bubble strip plot summarizing for each donor the corresponding group as defined by hierarchical clustering as shown in **(C)** and indicated on the x-axis (or the singletons for donors 025 and 032), his FCGR3A haplotype as well as the maximum percentage of specific lysis extracted from previous ADCC experiments using isolated CD56^+^ cells as shown in **Figure 3** encoded with colors and sizes as indicated in the legend.

Another approach consisted in the reanalysis of the previous flow cytometry data no longer on the scale of individuals but of single cells. We restricted the analysis to the NCR/KIR/KLR markers as well as CD16, CD57, and PD-1 and gated on the total CD56^+^ cells using the data from all the 30 donors, and right after we randomly selected 3,000 events from each donor all of which were finally concatenated together before dimensional reduction was performed through the t-SNE algorithm. Several clusters were identified in the resulting t-SNE map defined by the level of expression and different combinations of the markers analyzed as summarized on the heatmaps shown in **Figure 4A**. There was clearly one separated group of cells on the bottom-left of the t-SNE map mainly expressing CD16, KIR3DL1, and NKp46. Cells expressing CD16 and NKp46 but not KIR3DL1 were also grouped in another bigger cluster located on the right part of the t-SNE map, the latter also being characterized by a modest expression of CD57. A small separate island of cells expressing mainly high levels of NKG2C and CD57 could be detected in the top left. It is interesting to note that part of the cells expressing more strongly PD-1 were located in a region rather defined by a lack of expression of CD16, NKp46 and of all the KIRs, and by the low/intermediate expression of CD56, CD57, NKG2A, and NKG2C. We could finally distinguish a small group of cells strongly expressing CD56 but no CD16 at the bottom right on the t-SNE map, those CD56^hi^CD16^−^ cells also strongly expressed high levels of NKp46 and NKG2A.

The positive events for the markers NKp30, NKp44, and NKG2D seemed to be distributed homogeneously in all the regions of the t-SNE map, however, the diverse and complex combinations of expression of the other markers led us to define 18 distinct main clusters which grouped together the cells with a similar phenotypic profile (**Figure 4B**). For example, the cells grouped into the t-SNE cluster number 11, located in the islands at the bottom left of the t-SNE map, are all defined by the strong expression of CD16, NKp46, and KIR3DL1 (**Figure S10**), the ones grouped into the t-SNE cluster number 17 are rather defined by the lower expression of CD16, the lack of expression of all the KIRs as well as NKp46, and the strong expression of CD57. The number of events varied from 2,192 in the cluster 10 to 7,324 in the cluster 9. This preliminary step was the basis for the hierarchical classification of all the donors on the basis of the distribution of their CD56^+^ cells in the different t-SNE phenotypic clusters. The dendrogram resulting from the hierarchical clustering presented in **Figure 4C** depicts first of all a certain difficulty in grouping the donors into very distinct groups, with the heights of the shallow level nodes relatively high and a significant number of sub-clusters. After determining a reasonable cut-off at a distance of 32, five main groups and two singletons were identified. Interestingly, when we looked back at the t-SNE profile for the donors classified into group V, namely donors 015, 020, 024, 027, 029, 030, 031, 034, and 038, we noted the systematic absence or very low frequency of events in the islands at the bottom left of the t-SNE map which correspond to the phenotypic clusters number 10, 11 and 12 which had as a common factor the strong expression of KIR3DL1 marker. Furthermore we noted a higher frequency of cells in the phenotypic clusters number 17 and 18 which had as a common factor the strong expression of CD57 marker (**Figure S11**). In addition, when we looked more closely at the most similar donor pairs in other groups, such as 037/042 or 028/039, it appeared that their t-SNE phenotypic profiles were almost superimposable (**Figure S11**).

In order to make the comparison between individuals easier, we have represented the distribution of all CD56^+^ cells in the 18 t-SNE clusters and for each donor in a cumulative bar graph as shown in **Figure 4D**. We noted there the particularity of the donor 032 for which the cells are distributed very predominantly in clusters 9 and 13 which counted together for 63.6% of all the CD56-positive cells in this donor, were bordering on the map and whose particularity was a lack of expression of CD16, a weak expression of CD57 and NKp46 and a substantial expression of PD-1 (**Figures 4A, B**). The case of the donors 021 and 041 who belonged to group IV and accounted, respectively, for 41.3% and 36.2% of their CD56^+^ cells in the phenotypic t-SNE clusters number 13 and 14, which together are ultimately equivalent to what had been described for donor 032, with in addition the peculiarity of having a lower expression of NKG2A and a higher expression of PD-1 (**Figures 4A, B**). Apart from these isolated cases, and certain more or less obvious groupings such as between donors 019, 026, 035, and 040, or between donors 028, 033, 036, 039, 043, and 044, this approach allowed us to visualize the general diversity of CD56^+^ cells within and between individuals.

We finally compared the FCGR3A-158 haplotypes as well as the maximum ADCC activity measured with isolated CD56^+^ cells between the groups resulting from the hierarchical classification (**Figure 4E**). All the donors pooled in group III had a homogeneous cytolytic profile with high ADCC activity and there was no selection of a particular FCGR3A-158 haplotype. In group I, II, and V the ADCC activities were more heterogeneous but always without selection of a particular FCGR3A-158 haplotype. Finally, the ADCC activity measured for the three donors classified into group IV and the singleton 032, all these donors having been discussed above, was one of the weakest and this apparently independently of the haplotype. Nevertheless, the isolated CD56^+^ cells seem to be a good compromise as primary cells to study ADCC activities in the current experimental setting at least, and despite a great immune diversity whether at the individual level or at the level of the single-cell, the heterogeneity in the cytolytic responses observed was much less than when total PBMCs were used.

### Effects of Fc-Glycosylation of Trastuzumab on ADCC Activity of CD56^+^ Cells

Current monoclonal antibody production processes make the glycosylation of the Fc fragments of monoclonal antibodies subject to batch-to-batch variability, and this can have important consequences in terms of biological activity. The influence of IgG1 Fc galactosylation and sialylation on ADCC activity and binding to FcγRs has been investigated using different glyco-variants produced from anti-EGFR (10) and anti-HER2 trastuzumab (11) monoclonal antibodies by *in vitro* glycoengineering. In the present study, we were interested in the effects of the same samples used in a previous study (11), namely samples generated to obtain deglycosylated (*deglyc*), degalactosylated (*G0*), galactosylated (*G2*), as well as galactosylated followed by α2,3- (*ST3*) or α2,6-sialylation (*ST6*) variants of trastuzumab (*TRA*) on the ADCC activity of isolated CD56^+^ cells and in the presence of BT474 target cells.

**Figure 5:**
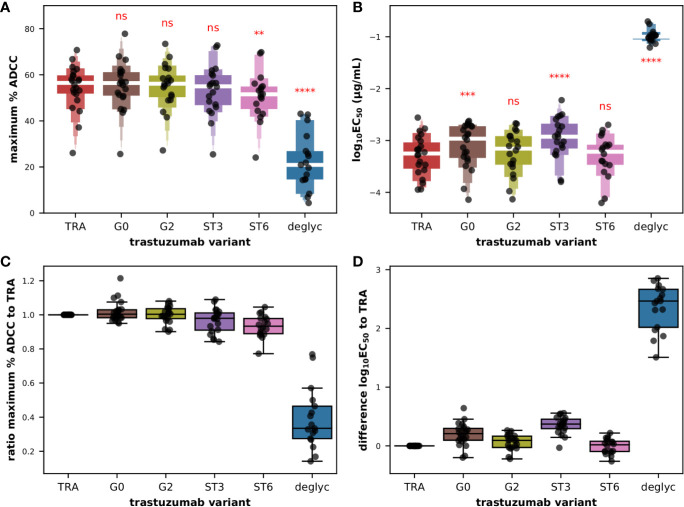
ADCC effector function of isolated CD56^+^ cells against BT474 target cells in the presence of glycoengineered low afucosylated variants of trastuzumab. Letter-Value box plots (with median in white) overlaid with strip plots summarizing **(A)** the maximum percent of specific lysis and **(B)** the EC_50_ values obtained with the indicated glycoengineered variant of trastuzumab (n = 22). Box plots overlaid with strip plots summarizing for each donor **(C)** the ratio of the maximum percent of specific lysis to the value obtained with unmodified trastuzumab, and **(D)** the difference between the common logarithm of the EC_50_ values obtained with the indicated glycoengineered variant and the value obtained with unmodified trastuzumab. Low afucosylated *G0*, *G2*, *ST3*, *ST6*, and deglycosylated (*deglyc*) antibody variants of the so-called unmodified starting material trastuzumab (*TRA*) have been described elsewhere (11). All the data used in this figure were obtained from experiments fully independent of the experiments presented in the previous figures. In **(A**, **B)**, the results of paired T-tests between the different groups and group *TRA* corrected for multiple-comparisons (Holm) after repeated-measures ANOVA are indicated in red. ****P ≤ 0.0001, ***P ≤ 0.001, **P ≤ 0.01, ns: not significant.

At first glance on the raw titration data, we noted an almost superimposition of the 4PL sigmoid curves obtained for all glyco-variants with the exception of *deglyc* for which the upper asymptote could never be reached and the value of EC_50_ was shifted toward lower affinity, and this was observed for all donors tested (**Figure S12**). The very low frequency of CD56^+^ cells, around 10% for a few donors, combined with variable yields of extraction of PBMCs from the buffy coats and of magnetic isolation of effector cells, allowed us to test the primary cells in number sufficient for the titration of several antibodies in 22 donors. Moreover in donors 031, 035, 037, and 044 overnight resting failed to maintain a sufficient number of cells to test the complete set of trastuzumab glyco-variants. However, the dataset was sufficient to demonstrate a statistically significant effect of the glycosylation of the Fc fragments of trastuzumab on the maximum ADCC activity (**Figure 5A**) as well as on the common logarithm of EC_50_ (**Figure 5B**) with F(5,60 = 97.2; P < 0.0001) and F(5,60 = 278.6; P < 0.0001), respectively, according to repeated measures ANOVA. As expected, deglycosylation had a statistically significant effect on maximum ADCC activity (corrected P < 0.0001) compared to the original trastuzumab antibody, dropping from a mean value of 54.4% ± 9.9 with *TRA* to 22.3% ± 12.0 with *deglyc* variant, according to *post hoc* pairwise T-tests with Bonferroni adjustments. Similarly, deglycosylation had to a dramatic effect on the functional avidity with the common logarithm of EC_50_ in µg/ml increasing from a mean value of −3.3 ± 0.4 with *TRA* up to −0.99 ± 0.12 with *deglyc* variant (corrected P < 0.0001). We also detected a significant effect of α2,6- (*ST6*; corrected P = 0.0076) but not α2,3-sialylation (*ST3*; corrected P = 0.299) on the maximum ADCC activity, with mean values of 50.8% ± 10.5 and 53.2% ± 11.0, respectively. Interestingly, the opposite was observed for the effect on the EC_50_, with mean values for the common logarithm of EC_50_ in µg/ml of −3.3 ± 0.4 (corrected P = 0.802) and −2.9 ± 0.4 (corrected P < 0.0001) for *ST6* and *ST3*, respectively. And while the maximal activity capacity ADCC did not change significantly after degalactosylation (*G0*; corrected P = 0.379) and galactosylation (*G2*; corrected P = 0.953), with mean values of the maximum percentage of specific lysis of 55.4% ± 10.4 and 54.9% ± 10.1, respectively, we found that degalactosylation but not galactosylation had a significant effect on functional avidity with mean values of the common logarithm of EC_50_ in µg/ml of −3.1 ± 0.4 (corrected P = 0.00011) and −3.2 ± 0.4 (corrected P = 0.050), respectively. When we look back on the raw data, we can appreciate all these subtle but significant differences, with globally a upper asymptote a little lower for the variant *ST6* (pink) than for the variants *G0*, *G2*, *ST3*, and the original antibody *TRA*, as well as a slight shift to the right of the fitting curves for the variants *ST3* (purple) and in some donors *G0* (brown), as seen on **Figure S12**.

The mean pairwise differences in the common logarithm of EC_50_ compared to that of *TRA*, that is to say the ratio of the EC_50_ values, were respectively of 0.196, 0.353, and 2.352 for *G0*, *ST3*, and *deglyc* variants as shown in **Figure 5D**. This goes in the same direction as the data of the binding to FcγRIIIA-158 F published previously with *G0* and *ST3* variants yielding relative binding levels of 86% and 60%, respectively (11). Here, *G0* and *ST3* variants demonstrated 80 and 58% of relative ADCC activity compared to untreated trastuzumab. Another interesting outcome was the complete absence of binding to the FcγRIIIA-158 F/V receptors (but not on FcγRIA) and a complete absence of ADCC activity using NK92 cells when *deglyc* was tested (11). In the current study with primary cytotoxic cells, the fitting curves were certainly shifted to the right (**Figure S12**) signifying a significant drop in functional avidity, but residual activity was still observed in all donors at higher concentrations of antibody, as reflected in a ratio of the maximum ADCC activity relative to that of *TRA* of 0.39, compared to ratios of 1.02 and 0.97 for *G0* and *ST3*, respectively (**Figure 5C**). Finally, the increase in binding of *G2* and *ST6* variants (relative binding of 120% and 146%, respectively) and in cytolytic experiments using NK92 cells (relative ADCC activity of 106% and 130%, respectively) observed previously (11) was not reproduced in the current experimental setting. Indeed, the mean pairwise differences in the common logarithm of EC_50_ compared to that of *TRA* were 0.062 and −0.008 for *G2* and *ST6* variants, respectively (**Figure 5D**). Furthermore, a subtle but significantly lower percentage of maximum lysis mean was obtained for the *ST6* variant with a ratio of the maximum ADCC activity relative to that of *TRA* of 0.93 compared to 1.00 for the *G2* variant (**Figure 5C**).

## Discussion

The initial objective of this study was to identify biomarkers in the population of circulating cytotoxic cells in healthy donors that may be linked to their cytotoxic activity, namely ADCC, in an experimental system where a target tumor cell-line express strongly HER2 and in the presence of anti-HER2 antibody trastuzumab. Rapidly we were confronted with the great diversity in the composition of the immune cells in the blood of the 30 healthy donors tested as summarized in **Figure 1**. Each donor had an almost unique phenotypic imprint, whether in the distribution of the main populations of immune cells such as T and B cells, monocytes, dendritic, and NK cells, or in the distribution of the main NK cell subsets. It should also be noted in this case that the exact role of each of these subsets of NK cells, as well as their origin and the maturation pathways they can follow, are still under debate (39) and is beyond the scope of this study. Nevertheless, the CD56^hi^ NK cells, also named CD56^bright^ elsewhere, are generally considered to be the precursors of the CD56^dim^ NK cells, and have usually been described as cytokine producing and regulatory cells, compared to CD56^dim^CD16^+^ cells which demonstrate more cytolytic activity (67). This inter-individual diversity was even more remarkable when we compared the expression of certain cytotoxic markers such as CD57, NKG2A, and KIR3DL1. Due to this high degree in diversity the use of primary cells, in particular extracted from the blood of healthy donors, in robust bioassays appears problematic to implement in a quality control context mostly due to the fact that this variability will likely not allow us to detect minor changes in product quality. Therefore, although primary cells might reflect the *in vivo* situation better than cell lines used in potency assays used in quality control, they can be expected not to be suitable in most cases due to the inherent variability resulting in low robustness of the system. However, it appeared to us as a valid approach to understand the parameters that are critical for the identified biological function and investigate the responses to quality attributes of manufacturing such as Fc glycosylation of IgG-type antibodies in a more robust assay system. We therefore characterized the primary cells further with regard to biomarkers previously reported to be involved in ADCC and their possible correlation to the cytotoxic activity.

The influence of the FCGR3A-158 haplotype was not conclusive when analyzing the maximum ADCC activity of whole PBMCs against the target cell line BT474 strongly expressing HER2 in the presence of trastuzumab due to the limited number of donors homozygous for the FCGR3A-158 V allotype as shown in **Figure 2**. However, we did observe a trend for better functional avidity in donors with one or two copies of the FCGR3A-158 V gene variant which makes us hypothesizing that all the cytotoxic cells potentially involved in ADCC in the current HER2 system have the necessary intrinsic capacities, i.e., the cytotoxic granules and the mechanisms to release them, independently of the FCGR3A-158 haplotype, whereas the latter likely being important for the affinity of FcγRIIIa for trastuzumab at the molecular level. It should be noted that we focused on the genotype of FCGR3A-158 but other SNPs and copy number variations of functional importance had been discovered for FcγRIIIa and other genes of the FcγR family, such as for the KIR family (68, 69), therefore, a more global analysis could certainly provide more information as to the influence of the FCGR polymorphisms on ADCC activity.

We then tried to establish correlations between the flow cytometry data and those of the ADCC experiments, as well as classification of the donors on the basis of all these data, with little success certainly due to the combination of a low number of individuals involved in this study and of the immense phenotypic diversity of their cytotoxic cells. However, we established two fairly significant correlations, on the one hand between the functional avidity and the frequency of CD56^hi^CD16^−^ NK cells, and on the other hand between the maximum specific lysis values and the overall frequency of total CD56^+^ cells in PBMCs. The first relationship suggested that the functional avidity increased with the fraction of CD56^int^CD16^hi^ combined with CD56^lo^CD16^−^ in NK cells, which is the complementary of CD56^hi^CD16^−^ cells. In other words the more CD56^lo^ NK cells together with NK cells expressing FcγRIII present in the PBMCs the more potent the ADCC. The second relationship was not completely new and has already been raised by another group that have shown a significant correlation between the frequency of CD56^+^CD16^+^ effector lymphocytes among PBMCs and ADCC induced by *in vitro* addition of trastuzumab in patients with operable breast cancer overexpressing HER2, before and after therapy with trastuzumab (70). Therefore, we later focused on the analysis of the cytolytic activity of isolated CD56^+^ cells which include not only NK cells but also NKT, γδ-T cells and particular subsets of T lymphocytes, dendritic and monocytic cells (26).

The meaningful phenotypic heterogeneity was also observed at the level of the CD56-positive population both from the point of view of the composition in NK, CD56^+^lineage^+^TCRγδ-1^−^ and γδ-T cells as well as the expression of FcγRIII and of various cytotoxic markers, in particular NKp46, NKG2A, and CD57, as shown in **Figure 3**, which could explain in part the differences in ADCC activity observed between donors with the use of whole PBMCs. We also noticed a marked standardization in the frequency of CD56^+^ cells expressing NKG2D, as well as globally a higher ratio of cells expressing the PD-1 exhaustion marker than in total PBMCs. These observations should be given special attention in a follow-up study. Nevertheless, we observed a sort of homogenization of the ADCC activity of CD56^+^ cells measured both at the level of the maximum specific lysis activity and of the EC_50_ value independently of the FCGR3A-158 haplotype, with a significant increase in functional avidity compared to PBMCs. It thus appeared that the concentration of ADCC-competent CD56^+^ cytotoxic cells, and perhaps the elimination of some regulatory cells found in total PBMCs, has made it possible to increase the functional avidity in ADCC reaction, at least in the current experimental setting, and this clearly independently of the FCGR3A-158 haplotype and despite the immune diversity between the individuals. However, this did not facilitate the classification of donors, on the contrary, in view of the reduction in phenotypic and functional differences.

The analytical approach of dimensional reduction through t-SNE at the scale of the single cell which we then used on CD56^+^ cells and by restricting ourselves essentially to the markers of the NCR/KIR/KLR repertoire as well as CD56, CD16, CD57, and PD-1 allowed to better classify the donors according to the frequency of their cytotoxic cells expressing particular combinations and various intensities of markers grouped into 18 major clusters as summarized in **Figure 4**. We could for example distinguish particular subsets of cells such as those that express CD16, KIR3DL1, and NKp46, others with a strong expression of PD-1 but no expression of CD16, NKp46 and KIRs and low expression of CD56, CD57, NKG2A, and NKG2C, others expressing NKG2C and CD57, and finally CD56^hi^CD16^−^ cells expressing high levels of NKp46 and NKG2A. The functional study of these subsets would require cell sorting by flow cytometry and a substantial number of PBMCs in order to be able to carry it out. Nevertheless, 3 donors (021, 022, and 041) could be grouped together after hierarchical clustering on the basis of the frequency of their cytotoxic cells in the different phenotypic clusters, and these all demonstrated minimal ADCC activity. On the verge of coincidence, the implication of the absence of CD16, together with the very weak expression of CD57 and NKp46 and/or the substantial expression of PD-1 in the large proportion if not the majority of the cells which compose the CD56^+^ population in these donors will remain to be confirmed with a larger number of donors with a similar phenotypic profile. But it is more difficult to explain the low ADCC activity measured for CD56^+^ cells from donors 016, 019, 020, 031, 040, and 042 on the basis of the t-SNE profiles, for which fine and complex combinations of several markers could come into play.

The accumulation of evidences throughout these experiments has comforted us in the choice of using purified CD56^+^ cells and not whole PBMCs or even pure NK cells as an ideal primary cell material for the final comparative experiments of this study, namely of ADCC activity of *in vitro* glycoengineered variants of trastuzumab. We did not expect to see large changes in biological activities between these glyco-variants, with the exception of the deglycosylated variant, as summarized in **Figure 5**, but precisely to test the limit of this experimental system by knowing the various parameters of variability in primary cytotoxic cells due to the immune diversity of healthy donors as described earlier. Nevertheless, the data presented in this study agreed to some extent with the biochemical characterization data and cytolytic activity using the NK92 cell lines published previously (11). Indeed, we observed a significant decrease in functional avidity with the *G0* and *ST3* variants compared to *TRA*, of the same order of magnitude as their relative binding to FcγRIIIA-158. But some questions remain and, although a total loss of binding of the deglycosylated variant to the receptor as well as of the cytotoxic activity of the cell line NK92 in the presence of *deglyc* had been noted previously, here a residual activity was indeed measured in all the donors when used at the highest concentrations. This might be explained by the fact that biochemical binding assays to characterize the interaction of FcγRs and deglycosylated Fc-portions of IgG-type antibodies commonly do not account for avidity effects using monomeric receptors in solution [see, e.g., (71, 72)], which are inherent in cellular systems and might be manifested here at very high concentrations of trastuzumab.

Finally, the enhanced binding of *G2* and *ST6* variants and increased cytolytic activity of NK92 cells observed previously in the presence of these two variants was not reproduced in the current study using CD56^+^, and even more, we observed a subtle but significant decrease in the maximum lytic activity of CD56^+^ cells in the presence of the *ST6* variant. Interestingly, this last finding is in line with the previous notion that alpha 2,6-linked sialylation of IgG1 Fc-glycans can negatively impact ADCC activity (73). One hypothesis could be that an avidity plateau has been reached with those primary cells, whose EC_50_ for trastuzumab was significantly lower than with NK92 cells (18), and which does not allow us to have an optimal amplitude margin in the measurement of improved functional avidities unlike with NK92 cells. We should take into consideration that NK92 is an immortalized cell-line engineered to express FcγRIIIA, introducing different functional biases which are still not fully understood. But although we have demonstrated the usefulness and the feasibility of an *ex vivo* system with the use of a particular population of primary cells isolated from the blood of healthy donors, i.e., CD56^+^, in particular for a comparative study of the effect of various glyco-modifications in the structure of the Fc region of trastuzumab, this system shows limits which are not necessarily due to the diversity between individuals. It remains to be evaluated whether this approach is valid for other antibodies, e.g., cetuximab, as well as for other functional assays such as ADCP, but this study could already make it possible to take a new step in the discussion of the biological effects of certain modifications in the structure of therapeutic antibodies in a clinical context. While glycoengineering may improve the binding affinity of antibodies to FcRs as demonstrated *in vitro* at the molecular level, and although this may be directly related to improved functional avidity as shown in an ADCC bioassay using NK cell lines such as NK92, they might not be able to show improvement in an *ex vivo* system utilizing primary cells. This might be due to the fact that an *ex vivo* system could be oversized for a more precision-aimed quality control study and would add a layer of complex biological parameters that are difficult to account for and for which the non-modified antibody might already demonstrate maximum potency.

## Materials and Methods

### Peripheral Blood Mononuclear Cells

Buffy coats from healthy blood donors (**Table S2**) provided by the Blood Donation Center from the University Hospital of Basel (Switzerland) after obtaining informed consent and data anonymization in accordance with the Swiss Federal regulations. PBMCs were isolated by density centrifugation of leukocytes using Ficoll-Paque® PLUS (GE Healthcare) and LeucoSep tubes (Greiner Bio-one) according to the instructions of the manufacturers. The mononuclear cell layer was carefully removed from the interface and washed twice with PBS (Gibco). PBMC suspensions were prepared at a concentration of 20 × 10^6^ cells/ml in 90% fetal bovine serum (Gibco), and 10% DMSO Grade culture (Sigma-Aldrich), aliquoted into 1-ml cryovials and frozen using CollCell® at −80°C. After 24 h, the PBMCs were transferred to a liquid nitrogen freezer and stored in the vapor phase, i.e., ≤ −150°C (74, 75). For resting and short-term culture, PBMCs were maintained in *PBMC medium* consisting of RPMI-1640 (ATCC^©^ modification) supplemented with 10% heat-inactivated fetal bovine serum (Gibco), 100 U/ml penicillin and 100 μg/ml streptomycin (Gibco) at 37°C under 5% CO_2_ in a humidified incubator (76).

### Genotyping of *Fcgr3a* Single-Nucleotide Polymorphism

Genomic DNA was extracted from PBMCs using the QIAamp DNA Mini Kit (Qiagen) according to the manufacturer’s instructions. Genotyping of FcγRIIIA at amino acid position 158 (FCGR3A.Phe158Val or rs396991 A>C as represented on plus chromosomal strand, complemented because gene is on minus strand) variants was performed on genomic DNA by Droplet Digital™ PCR (ddPCR) as described elsewhere (77). Briefly, ddPCR was performed using a QX100 droplet generator with QX200/QX100 Droplet Generation Oil for Probes, DG8 Cartridges and DG8 Gaskets, T-100 thermal cycler, QX100 droplet reader with ddPCR Droplet Reader Oil and QuantaSoft software (v1.7.4.0917), all from Bio-Rad. Primers and VIC/FAM probes targeting *Fcgr3a* nucleotide 559 were generated by ABI/Thermo-Fisher Scientific as the TaqMan™ SNP Genotyping Assay 40X (human, ID C:25815666_10). Final reaction mix consisted of 900 nM of each primer, 250 nM of each probe, 1X ddPCR Supermix for Probes (no dUTP; Bio-Rad) and 5 ng of genomic DNA diluted in UltraPure™ DNase/RNase-Free Distilled Water (Invitrogen). Each DNA sample was tested in triplicate. Thermal cycling conditions utilized were 5 min at 95°C, 40 cycles of 30 s at 95°C and 1 min at 58°C, followed by 5 min at 4°C, 5 min at 98°C, and a final hold at 12°C, with a ramp rate of 2.5°C/sec and a lid heated at 105°C. FCGR3A-158 haplotype was determined by calculating the ratio of FCGR3A-158 F [rs396991(A)] to FCGR3A-158 V [rs396991(C)] allele counts in each sample. Expected ratios for homozygous wild-type FCGR3A-158 F/F was 2:2, resulting from identification of both the FCGR3A and closely related FCGR3B. Expected ratios for heterozygous FCGR3A-158 F/V and homozygous FCGR3A-158 V/V were 1:3 and 0:4, respectively. Ratios were calculated by comparing the total copy number counts for rs396991(A) and rs396991(C) alleles, as determined by the total number of positive droplets in the FAM and VIC channel, respectively.

### Flow Cytometry

Cryopreserved PBMCs were thawed rapidly in a water bath at 37°C, and washed in PBMC medium. Cells were then resuspended in PBMC medium, filtered through a 40-μm cell strainer (Falcon), and incubated at 37°C under 5% CO_2_ in a humidified incubator for 18 hours (74). After resting, the number of viable cells was determined using Trypan blue (Sigma-Aldrich). Cells were washed and suspended in cold *FACS buffer* consisting of PBS without Ca^2+^/Mg^2+^ (Gibco) supplemented with 2% fetal bovine serum (Gibco), 2 mM EDTA (Sigma) and 0.05% NaN_3_ (iNtRON), and incubated for 15 min with Human TruStain FcX™ FcR Blocking Solution (BioLegend) and then stained for 30 min at 4°C with the antibody panels defined in **Table S1** (78, 79). *Panel 1* was used for the characterization of FcγRs and FcRLs on cytotoxic cells, *Panel 2* for the determination of the NCR/KIR/KLR repertoire, and *Panel 3* for the immunophenotyping of the main immune populations in the PBMCs. Preliminary titration experiments were carried out to determine the specificity and the optimal concentration of each antibody (data not shown). The antibody panel cocktails were prepared in final volume of 50 μl per samples in FACS buffer containing 10 μl of Brilliant Stain Buffer PLUS (BD Biosciences) and 1 μl of Fixable Viability Dye (FVD) eFluor 780 (eBioscience). After staining, samples were washed twice in FACS buffer and fixed at room temperature for 20 min in Fixation Buffer 1X (BioLegend). Finally, cells were washed twice and resuspended in 200 μl of FACS buffer. UltraComp eBeads Compensation Beads (Invitrogen) were used according to manufacturer’s instructions to prepare compensation controls with fluorescently conjugated antibodies used in the experiments. Flow cytometry analysis was carried out on a BD LSRFortessa™ cell analyzer (BD Biosciences) equipped with UV (355 nm), violet (405 nm), blue (488 nm), yellow-green (561 nm), and red (640 nm) lasers. Instrument cleaning and Cytometer Setup and Tracking (CST) were run on a regular basis to ensure optimal cytometer performance. Up to 300,000 events were acquired using BD FACSDiva Software (v8.0.1). The computation of the compensation matrices as well as the analysis of FCS3.1 files were performed in FlowJo™ (v10.6.1; BD Biosciences). Dead cells, debris, and cell aggregates were excluded, and the populations of interest defined using the gating strategies detailed in **Figure S1**.

### Isolation of CD56^+^ Cells

CD56^+^ effector cells were isolated from PBMCs using EasySep™ Human CD56 Positive Selection Kit II (StemCell Technologies) according to the manufacturer’s instructions. Briefly, PBMCs were thawed rapidly in a water bath at 37°C, washed in PBMC medium, and treated with 100 U/ml of DNaseI grade I (Roche) for 15 min before filtering through a 40-μm cell strainer (Falcon). CD56-expressing cells were targeted with antibody complexes recognizing CD56 and dextran-coated magnetic particles. The cocktail also contains an antibody to the human Fc receptor to minimize nonspecific binding. Labeled cells were separated using an EasySep™ Magnet (StemCell Technologies). *MACS buffer* consisting of PBS without Ca^2+^/Mg^2+^ (Gibco) supplemented with 2% fetal bovine serum (Gibco) and 1 mM EDTA (Sigma) was used throughout the procedure. Desired cells remained in the tube while unwanted cells were poured off. Cell purity in the CD56-positive fraction was >90% as determined by flow cytometry (data not shown). Cells were then resuspended in PBMC medium and incubated at 37°C under 5% CO_2_ in a humidified incubator for 18-h resting (74).

### Cell Line and Cell Culture

The human breast cancer BT474 cell line (ATCC^©^ HTB-20™) was obtained from American Type Culture Collection. The cells were maintained in DMEM/F-12 medium with GlutaMAX (Gibco) supplemented with 10% heat-inactivated fetal bovine serum (Gibco), 5 μg/ml insulin solution from bovine pancreas (Sigma), 10 mM HEPES (Gibco), 100 U/ml penicillin, and 100 μg/ml streptomycin (Gibco). Cells were grown at 37°C under 5% CO_2_ in a humidified incubator. Prior to experimentation, cells (passages 8–18) were cultured in a 75 cm² flask to approximately 70 to 80% confluence. Cells were detached with 1X Trypsin-EDTA 0.25% (Gibco), and the expression of HER2 was monitored using an anti-CD340 (clone 24D2) and the corresponding isotype control (both from Miltenyi Biotec) using flow cytometry (data not shown).

### *In vitro* ADCC Assay

Thawed PBMCs or primary CD56^+^ cells were used as effector cells after a 18-h resting period. The cytolytic activity was analyzed in a EuTDA cytotoxicity assay (PerkinElmer) according to the manufacturer’s recommendations. Briefly, BT474 cells were harvested and loaded with the fluorescence enhancing ligand DELFIA® BATDA (PerkinElmer) for 20 min at 37°C. The number of viable cells was determined using Trypan blue (Sigma-Aldrich). After three wash cycles, 5 × 10^3^ target cells per well were seeded in 96-V-bottom culture plates in the presence of trastuzumab (subcutaneous formulation of Herceptin® SC, F. Hoffmann-La Roche Ltd, Basel, Switzerland) or glycoengineered variants of the low afucosylated format of trastuzumab as described elsewhere (11) at concentrations ranging from 1 × 10^−7^ to 1 × 10^0^ μg/ml, in PBMC medium. The precise antibody concentrations actually tested were 1 × 10^0^, 1 × 10^−1^, 1 × 10^−2^, 1 × 10^−3^, 1 × 10^−4^, 1 × 10^−5^, 1 × 10^−6^, and 1 × 10^−7^ μg/ml, including PBMC medium without antibody control (data not shown). After 15 min of pre-incubation, effector cells were added at the indicated effector to target (E:T) ratio, defined as ratio of whole PBMC (**Figure 2**) or isolated CD56^+^ cell (**Figures 3** and **5**) number to BT474 cell number, in a final volume of 200 μl. Duplicate wells were set up for each E:T ratio and antibody concentration condition. After 3.0 h (glyco-engineered variant experiments) or 2.5 h (all other ADCC experiments) co-culture at 37°C under 5% CO_2_ in a humidified incubator, plates were centrifuged at 500 × g for 5 min, and 20 μl of supernatant was mixed with 200 μl DELFIA® Europium Solution (PerkinElmer) in a 96-well clear bottom plates, followed by 15-min incubation at room temperature on a plate shaker. Time-resolved fluorescence was measured in RFU (Relative Fluorescence Units) using excitation at 345 nm, emission at 615 nm and cutoff at 590 nm, and a 1.0-ms integration time. Measurements were performed on a Spectramax M5 plate reader and SoftMax Pro Software (v6.5.1; Molecular Devices). The percentage of specific lysis was calculated according to equation (1), in which *spontaneous release* refers to the TDA release of target cells incubated without effector cells, and the *maximum release* is the TDA release of target cells in the presence of 25 μl of Lysis Buffer (PerkinElmer).

(1) $$\text{percent}\;\text{specific}\;\text{lysis}=100\times \frac{\left(\text{experimental}\;\text{release}\;-\;\text{spontaneous}\;\text{release}\right)}{\left(\text{maximum}\;\text{release}-\text{spontaneous}\;\text{release}\right)}$$

LMFIT (v1.0.0) (80) was used to analyze dose-response data using a 4-parameter logistic (4PL) regression sigmoidal dose-response fitting curve according to equation (2), with *x* the log_10_-transformed antibody concentration values, *y* the percent specific lysis values obtained in equation (1), *bottom* and *top* the values of the lower and upper asymptote, respectively, also called *minimum* and *maximum percent of specific lysis*, respectively in the text, EC_50_ the value of the 50% effective concentration, and *H* the value of the Hill slope.

(2) $$y=\text{bottom}+\frac{\text{top}-\text{bottom}}{1+10^{({\text{log}}_{10}\;EC_{50}-x)\times H}}$$

### Statistical Analysis and Data Visualization

All raw data in CSV format were analyzed with pandas (v1.0.1) (81). Pandas was also used for pairwise correlation analyses (Kendall). All plots were built using matplotlib (v3.1.3) (82) and seaborn (v0.10.1). SciPy (v1.4.1) (82) was used for linear regression, paired and unpaired T-tests (two-tailed), hierarchical clustering (Ward method) and dendrogram representation. scikit-learn (v0.22.1) (83) was used for PCA. pingouin (v0.3.3) (85) was used for one-way ANOVA followed by pairwise Tukey-HSD *post hoc* tests, mixed-design ANOVA (with Greenhouse-Geisser correction) followed by pairwise T-tests with Benjamini/Hochberg FDR correction, and repeated measures ANOVA (with listwise deletion of missing values) followed by pairwise T-tests with step-down method using Bonferroni adjustments and a pairwise deletion of missing values. In all the figures, the level of statistical significance expressed as P values are reported as followed: **** : P ≤ 0.0001, *** : P ≤ 0.001, ** : P ≤ 0.01, * : P ≤ 0.05, and not significant (*ns*) : P > 0.05). Mean values ± standard deviation with Delta Degrees of Freedom of 1 are used throughout the text. Python (v3.7.6) was used to execute all the scripts. Dimensionality reduction of the flow cytometry data was performed with the t-Distributed Stochastic Neighbor Embedding (t-SNE) algorithm integrated natively into FlowJo™ (v10.6.1; BD Biosciences). Briefly, raw data were cleaned up and a downsample gate created on CD56-positive cells (3,000 events for each donor). Compensated parameters of interest were selected and scaled using an ArcSinh transformation. The data thus prepared for the 30 electronically barcoded donors were then all concatenated in a single file and submitted to the t-SNE algorithm with 1,000 iterations, perplexity of 30 and learning rate of 6,166. The data were further explored by manual gating on clusters that were phenotypically similar. Finally Inkscape (v0.92) was utilized to combine individual SVG layouts from FlowJo and matplotlib in a single figure.

## Data Availability Statement

All data files and scripts used for analyzes are accessible via the following GitHub repository: https://github.com/sbwiecko/ADCC_primary_CD56_manuscript/. The flow cytometry data generated for this study are available in FlowRepository (https://flowrepository.org) as experiment IDs FR-FCM-Z2HV, FR-FCM-Z2WZ, FR-FCM-Z2WY, FR-FCM-Z2W3, FR-FCM-Z2W4 and FR-FCM-Z2W6.

## Ethics Statement

Ethical review and approval was not required for the study on human participants in accordance with the local legislation and institutional requirements. The patients/participants provided their written informed consent to participate in this study.

## Author Contributions

SW and FC designed the study and wrote the manuscript. SW designed and performed the experiments, acquired data, performed data analysis, and prepared the figures and supplementary materials. CA, AO, and DG contributed to the study design and the discussion. All authors contributed to the article and approved the submitted version.

## Funding

This work was supported by Roche. The funder had the following involvement in the study: participation in study design, participated in collection, analysis, interpretation of data, the writing of this article and the decision to submit it for publication.

## Conflict of Interest

SW and DG received a scientific grant from Roche. CA and FC were employed by Roche. AO was employed by Genentech.

The remaining authors declare that the research was conducted in the absence of any commercial or financial relationships that could be construed as a potential conflict of interest.
