# Supplementary material for: Toward a Better Understanding of Bioassays for the Development of Biopharmaceuticals by Exploring the Structure-Antibody-Dependent Cellular Cytotoxicity Relationship in Human Primary Cells
Source: Front Immunol. 2020 Oct 29;11:552596. doi: 10.3389/fimmu.2020.552596 (PMC7658677; doi:10.3389/fimmu.2020.552596)
Supplement: Supplementary file 1 [file Presentation_1.pdf]

Supplementary Material

SUPPLEMENTARY TABLES

The current document includes the following Tables:

Table S1: antibodies used in the flow cytometry experiments in this study, i.e. the name of the antigen and the corresponding fluorochrome, clone, supplier and catalog number of the antibody. Of note, the KIR-NKAT2 antibody clone DX27 recognizes KIR2DL2, KIR2DL3 and KIR2DS2 (KIR2DL2/3/S2), and CD158 antibody clone HP-MA4 recognizes KIR2DL1 (CD158a), KIR2DS1 (CD158h), KIR2DS3 and KIR2DS5 (CD158g) (KIR2DL1/S1/3/5). The Lineage Cocktail 3 (lin 3) includes CD3 (clone SK7), CD14 (clone MφP9), CD19 (clone SJ25C1), and CD20 (clone L27) antibodies.

Table S2: detailed information about the donors included in this study, i.e. the FCGR3A-158 haplotype, sex (F for female and M for male) and age.

SUPPLEMENTARY FIGURES

The current document includes the following Figures:

Figure S1: clean-up and gating strategies for the flow cytometry experiments.

Figure S2: expression of cytotoxic markers in NK cells in each donor.

Figure S3: information regarding genotyping of *Fcgr3a* SNP.

Figure S4: additional ADCC data using primary total PBMCs as effector cells.

Figure S5: PCAs using data from NK and CD56-positive cells and from the main immune populations.

Figure S6: diversity in the expression of cytotoxic markers in $CD56^+lineage^+$ cells.

Figure S7: information regarding ADCC using isolated $CD56^+$ as effector cells.

Figure S8: heatmap summarizing the correlation between different parameters of $CD56^+$ cells.

Figure S9: PCAs using a restricted number of features and focusing on specific cytotoxic cell subsets.

Figure S10: histograms generated upon query gating on the dimensionality reduction analysis dot plot obtained in Figure 4B.

Figure S11: projection on the dot plot resulting from the tSNE analysis discussed in Figure 4.

Figure S12: additional ADCC data using isolated $CD56^+$ and glyco-engineered variants of trastuzumab.

Table S1. List of antibodies used in the flow cytometry experiments.

panels	antigen	fluorochrome	clone	supplier	catalog number
3	CD3	AF488	UCHT1	BD	557694
3	CD4	BV510	SK3	BD	562970
3	CD8	BV650	RPA-T8	BD	563821
3	CD11c	PE-Cy5	B-ly6	BD	551077
3	CD14	BUV395	MfP9	BD	563561
1,2	CD16	BV510	3G8	BD	563830
3	CD16	BV786	3G8	BD	563690
1	CD16b	PE	REA589	Miltenyi	130-109-145
3	CD20	PE-Cy7	2H7	BioLegend	302312
3	CD25	BV711	2A3	BD	563159
1	CD32	BV650	FLI8.26	BD	740572
3	CD38	BV421	HIT2	BD	562444
3	CD45RA	PerCP-Cy5.5	HI100	BD	563429
1	CD56	APC	REA196	Miltenyi	130-113-310
2,3	CD56	BUV737	NCAM16.2	BD	564447
2	CD57	BV605	QA17A04	BioLegend	393304
1	CD64	BUV737	10.1	BD	612776
3	CD123	APC	7G3	BD	560087
3	CD127	PE	HIL-7R-M21	BD	561028
2	CD158 (KIR2DL1/S1/3/5)	PerCP-Cy5.5	HP-MA4	BioLegend	339514
2	CD158e (KIR3DL1)	APC	DX9	Miltenyi	130-092-474
2	CD159a (NKG2A)	PE	REA110	Miltenyi	130-113-566
2	CD159c (NKG2C)	PE-Vio770	REA205	Miltenyi	130-120-449
3	CD197 (CCR7)	PE-CF594	150503	BD	562381
2	CD279 (PD-1)	PE-CF594	EH12.1	BD	565024
1	CD307c (FcRL3)	BV786	H5	BD	744240
1	CD307e (FcRL5)	BUV395	509F6	BD	749609
2	CD314 (NKG2D)	BV421	1D11	BioLegend	320822
2	CD335 (NKp46)	BV650	9E2/NKp46	BD	563230
2	CD336 (NKp44)	BV711	p44-8	BD	744303
2	CD337 (NKp30)	BV786	p30-15	BD	743172
1	FcRL6	BV421	2H3	BD	747797
3	HLA-DR	BV605	G46-6	BD	562845
2	KIR-NKAT2 (KIR2DL2/3/S2)	BUV395	DX27	BD	745607
1,2	Lineage Cocktail 3	FITC	lin 3	BD	643510
1,3	TCRγδ-1	APC-R700	11F2	BD	657706

Table S2. Detailed donor information.

donor	FCGR3A-158 haplotype	sex	age
015	F/F	M	20
016	F/F	M	51
017	F/F	F	25
018	F/V	F	26
019	V/V	M	74
020	F/F	F	32
021	F/F	M	54
022	F/V	M	47
023	F/F	M	56
024	F/V	M	27
025	F/V	M	52
026	F/F	F	30
027	F/F	F	55
028	F/F	F	28
029	F/V	M	47
030	F/V	M	54
031	F/V	M	47
032	V/V	M	72
033	F/V	M	49
034	F/F	M	32
035	F/V	F	42
036	F/V	F	30
037	F/V	M	44
038	V/V	F	62
039	F/F	M	22
040	F/F	F	35
041	F/V	F	30
042	F/V	F	30
043	F/F	M	64
044	F/V	M	56

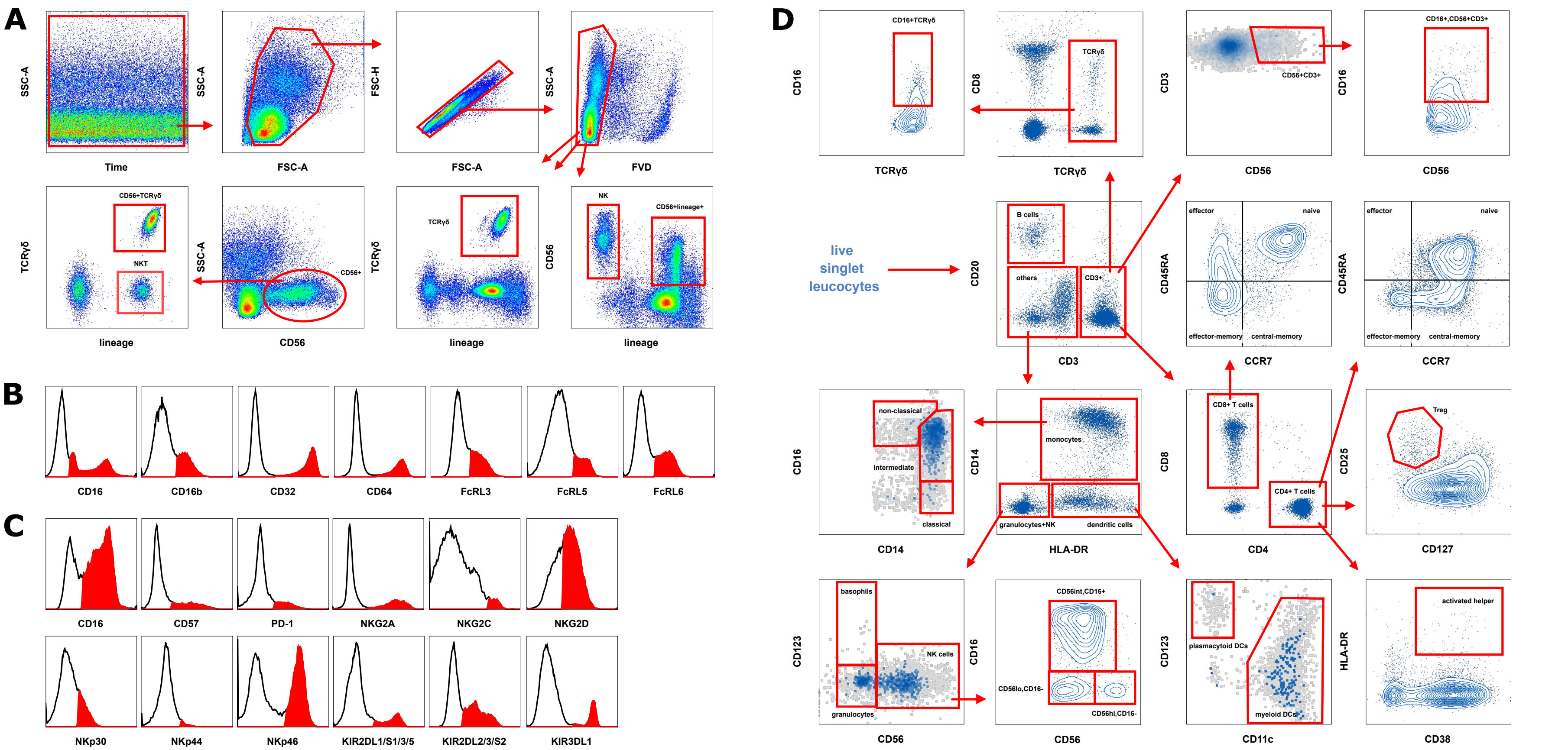

Figure S1: Clean-up and gating strategies **(A)** for the flow cytometry panels dedicated to the characterization of **(B)** the FcγR and FcRL receptors, **(C)** the NCR/KIR/KLR markers, and **(D)** the main immune cell populations. The gating in **(A)** differs depending on the analysis performed, i.e. on total living cells or on CD56^{+} cells. The populations considered positive for the indicated markers are filled in red on the histograms in **(B)** and **(C)**.

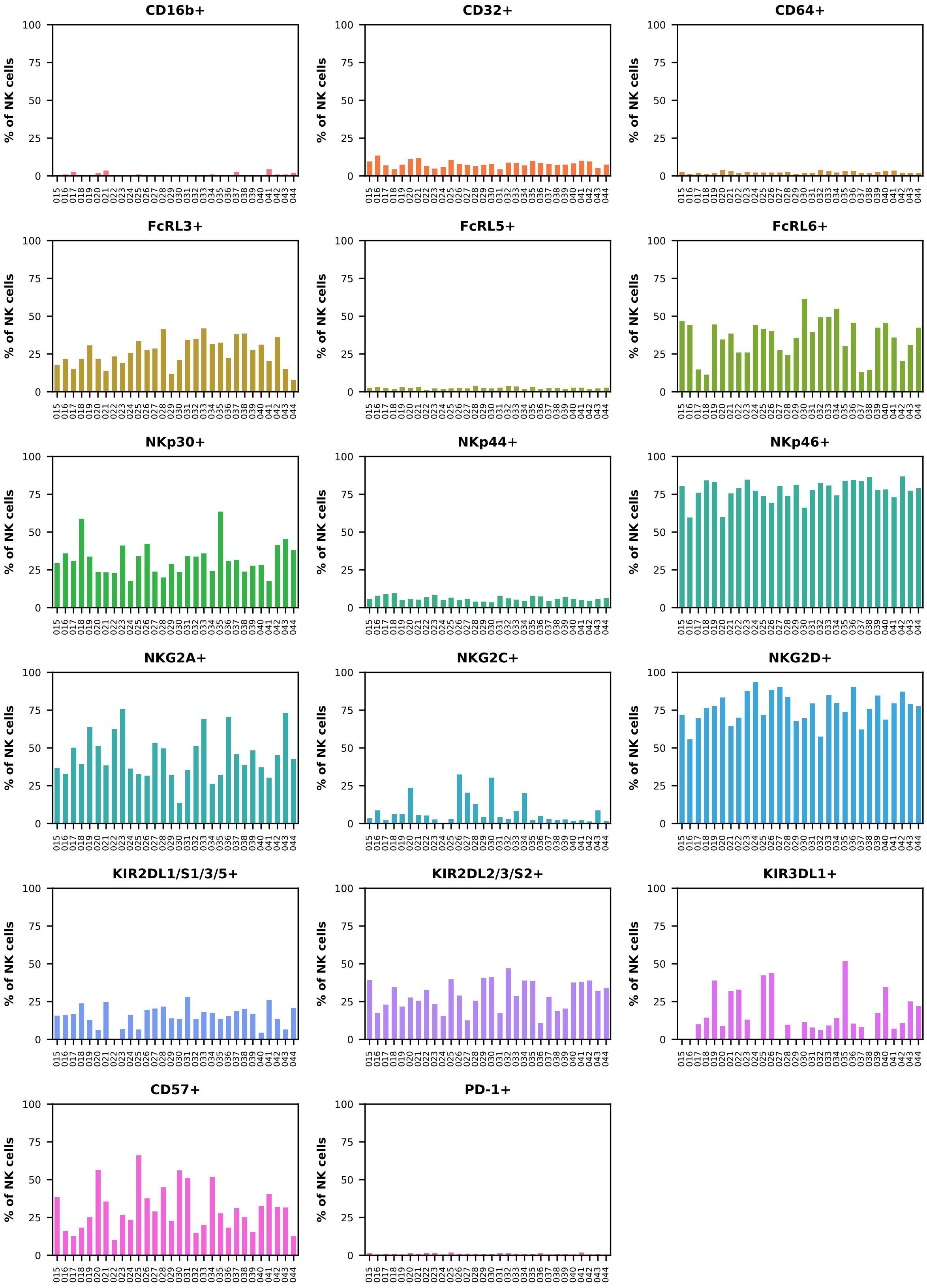

Figure S2: Individual bar plots showing the frequency of NK ($CD56^{+}lineage^{-}$) cells expressing the indicated marker in each donor as analyzed by flow cytometry (n=30).

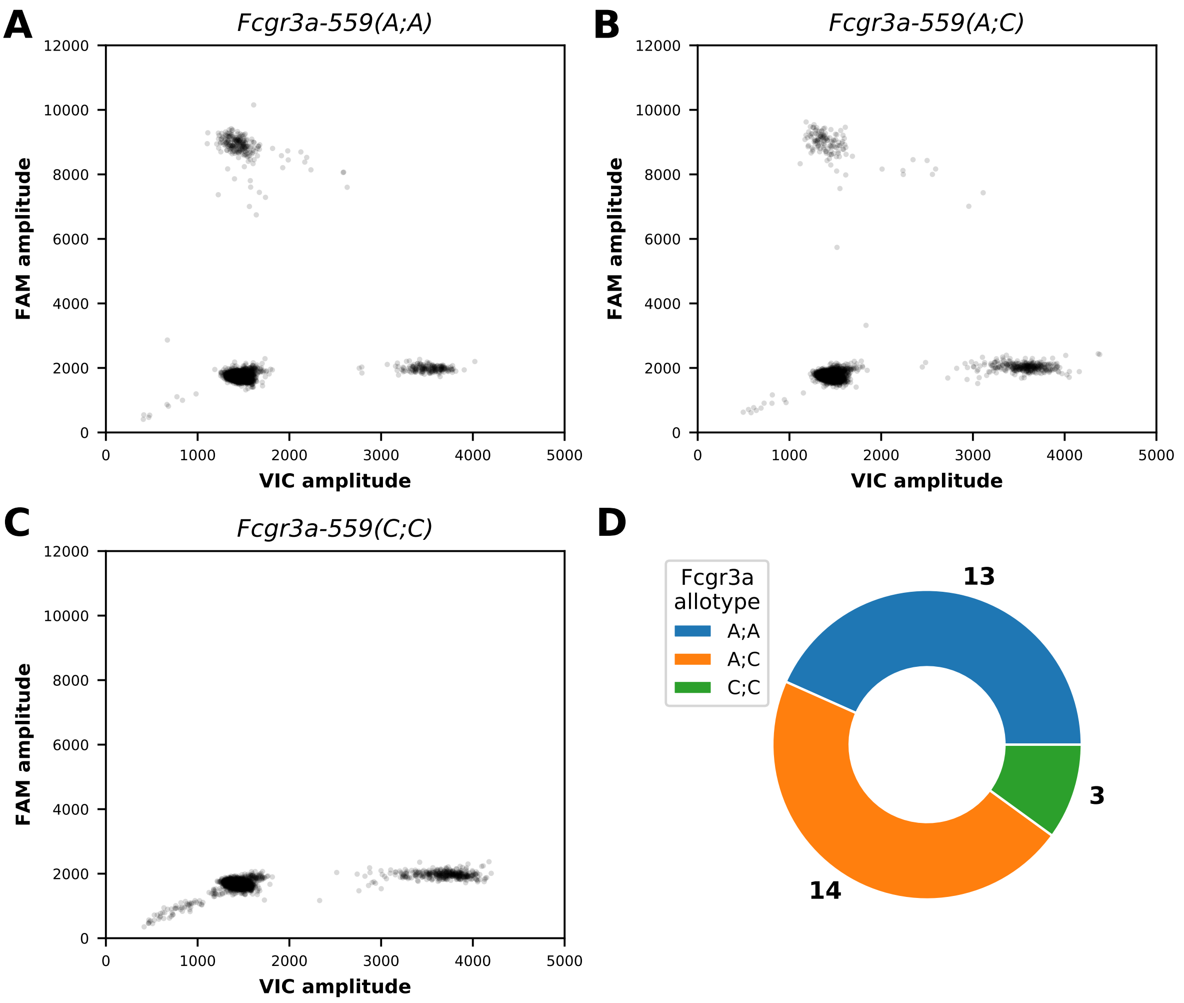

Figure S3: Additional information regarding the genotyping of *Fcgr3a* SNP used in this study. Representative dot plots of the raw data obtained using ddPCR for homozygous *A;A* (**A**), heterozygous *A;C* (**B**) and homozygous *C;C* (**C**) allotypes. (**D**) Pie chart summarizing the number of donors with the indicated *Fcgr3a* allotype.

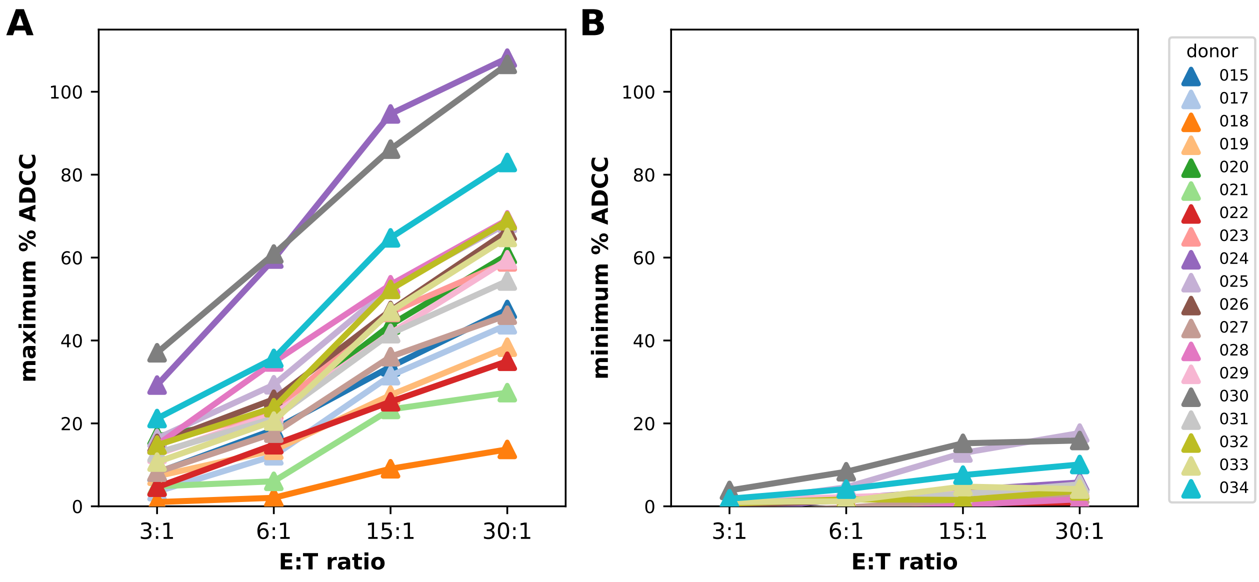

Figure S4: Additional ADCC data using primary total PBMCs as effector cells, BT474 target cells and trastuzumab. The maximum **(A)** and minimum **(B)** percent of specific lysis values were extracted from the 4-parameter sigmoidal dose-response fitting as described in Materials and Methods, for the 19 donors indicated in the legend and the E:T ratio indicated on the x-axis. The maximum and minimum specific lysis values are ultimately the specific lysis values respectively at the top and bottom plateau, also called higher and lower asymptotes, of the curve resulting from the 4PL regression that fits all data points at all concentrations tested. It can be noted that the bottom plateau is generally reached for trastuzumab concentrations around 1×10^{-5} μg/mL.

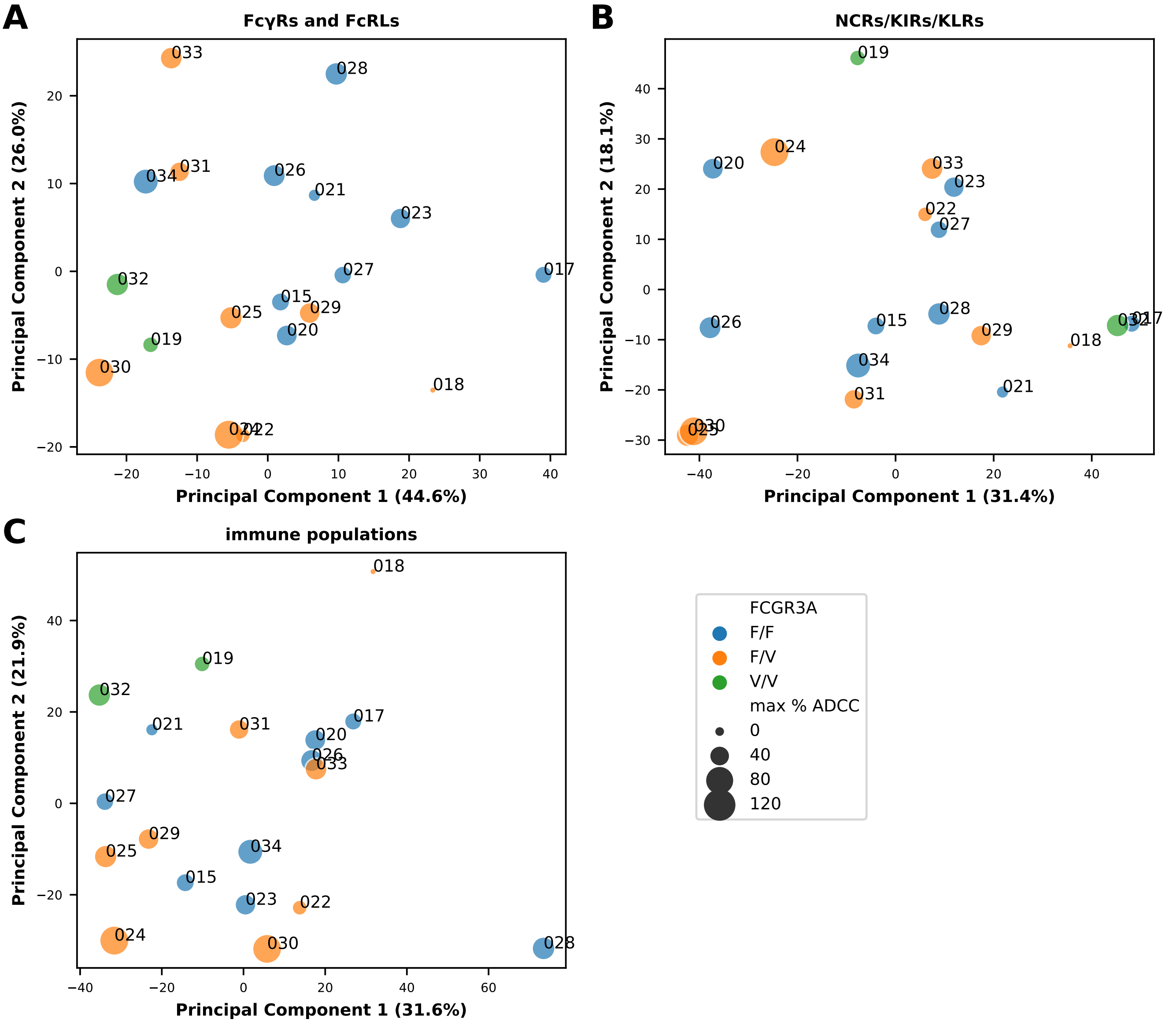

Figure S5: Bubble plots showing the results of PCA using **(A)** the expression of FcγRs and FcRLs in NK (CD56$^+$lineage$^-$) and CD56$^+$lineage$^+$ cells, **(B)** the expression of NCRs/KIRs/KLRs in NK (CD56$^+$lineage$^-$) and CD56$^+$lineage$^+$ cells, and **(C)** the composition of the main immune populations and their subsets. For each donor, a data point summarizes its FCGR3A haplotype and the value of the maximum percent of specific lysis obtained with total PBMCs used at an E:T ratio of 30:1, as shown in Figure 2 and Figure S4, encoded with colors and sizes as indicated in the legend. Values in parentheses show the percentage variance explained by each PCA axis.

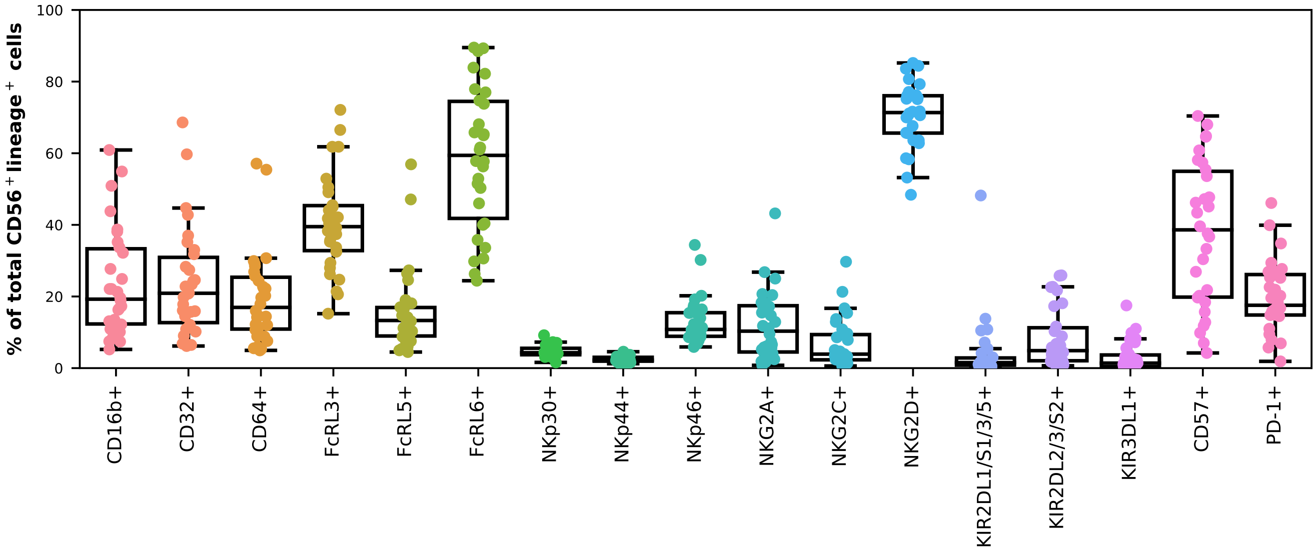

Figure S6: Diversity in the expression of cytotoxic markers in CD56$^+$lineage$^+$ cells in the blood of the healthy donors analyzed by flow cytometry. Box plots overlaid with strip plots summarizing the frequency of CD56$^+$lineage$^+$ cells, including NKT cells and subsets of T lymphocytes, expressing the indicated marker (n=30). This figure can be compared to Figure 1C, in which the same data set was used but the frequencies of NK (CD56$^+$lineage$^-$) cells expressing the different markers instead of CD56$^+$lineage$^+$ cells are shown, and to Figure 3D in which total CD56$^+$ were analyzed.

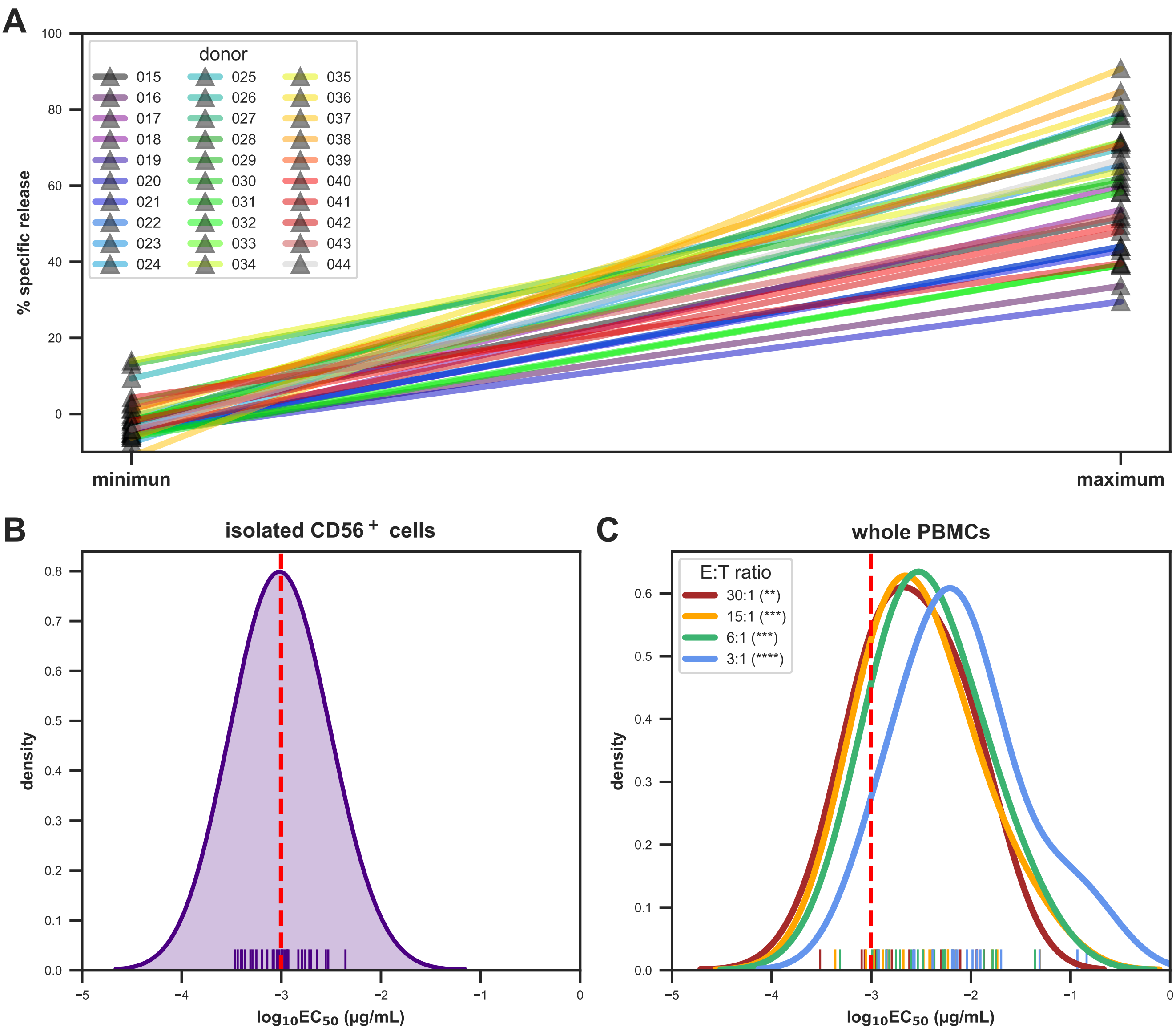

Figure S7: Additional information regarding the ADCC data using isolated CD56$^+$ as effector cells, BT474 target cells and trastuzumab. **(A)** Minimum and maximum percent of specific lysis represented on a before—after line plot as obtained using isolated CD56$^+$ cells and an E:T ratio of 5:1, and refering to data presented in Figures 3E-G. **(B)** Histogram of the Kernel Density Estimation overlaid with a rug plot summarizing the distribution of the EC_{50} values obtained with isolated CD56$^+$ cells (n=30) used at an E:T ratio of 5:1. Similarly and for direct comparison, **(C)** summarizes the distribution of the EC_{50} values obtained with whole PBMCs (n=19) used at different E:T ratios as indicated in the legend. In both plots, a red dashed line is drawn at the mean EC_{50} value obtained with isolated CD56$^+$ cells. The results of paired T-tests comparing the distribution of the common logarithms of the EC_{50} values obtained using PBMCs at different E:T ratios and CD56$^+$ effector cells in the 19 donors in common in both experimental settings are indicated beside the corresponding E:T ratio in the legend. The difference between the distributions of the EC_{50} values is even more statistically significant when we compare the 30 values in **(B)** to the 19 values in **(C)** at all E:T ratios using unpaired T-tests.

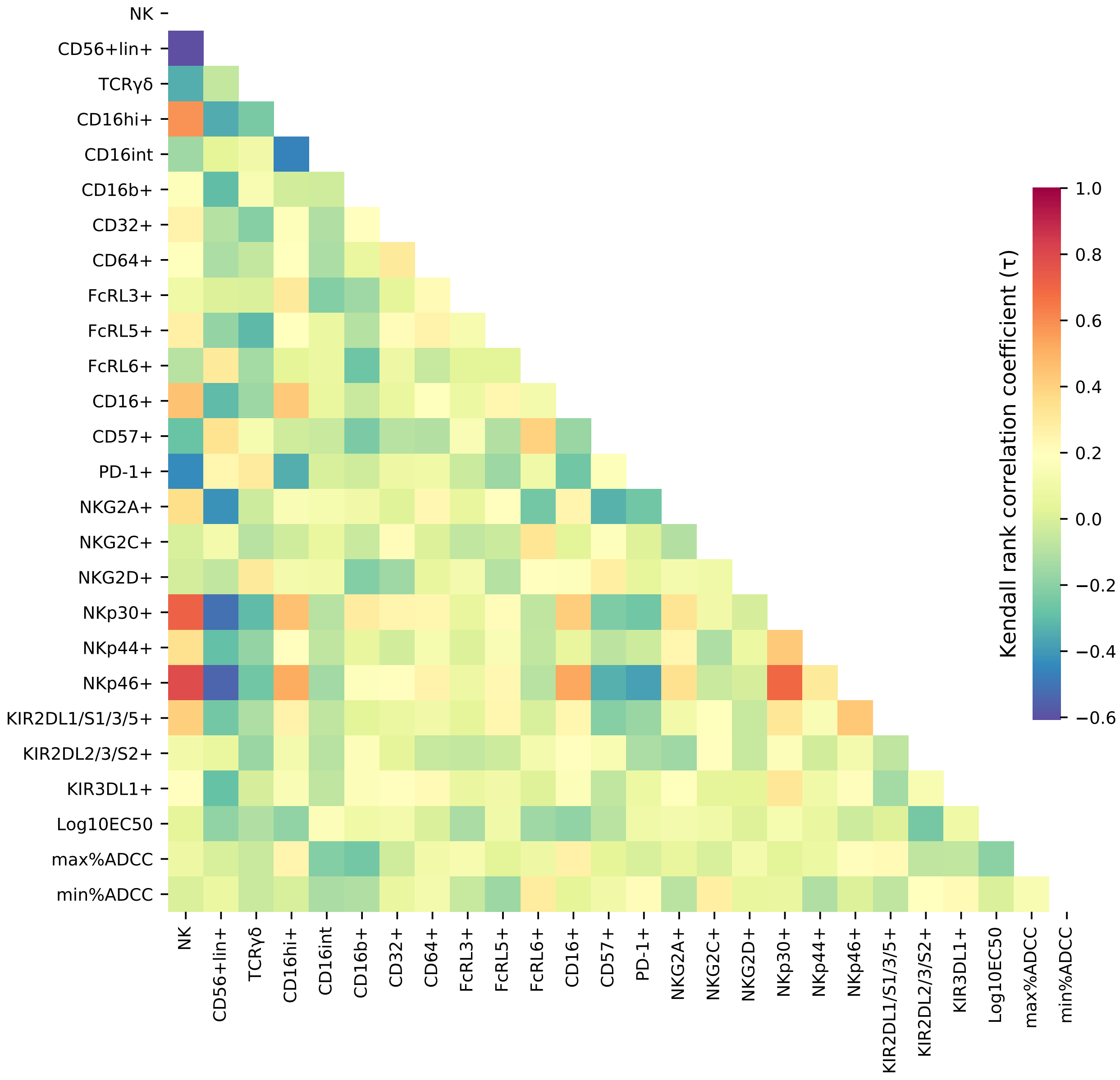

Figure S8: Heatmap summarizing the correlation coefficients (Kendall rank) between the frequency of NK cells ($CD56^{+}lineage^{-}$), $CD56^{+}lineage^{+}$ and γδ-T cells, the level of expression of FcγR, FcRL, NCR, KLR and KIR markers on total $CD56^{+}$ cells and the ADCC parameters obtained using the isolated $CD56^{+}$ cells as effector cells, BT474 target cells and trastuzumab (n=30).

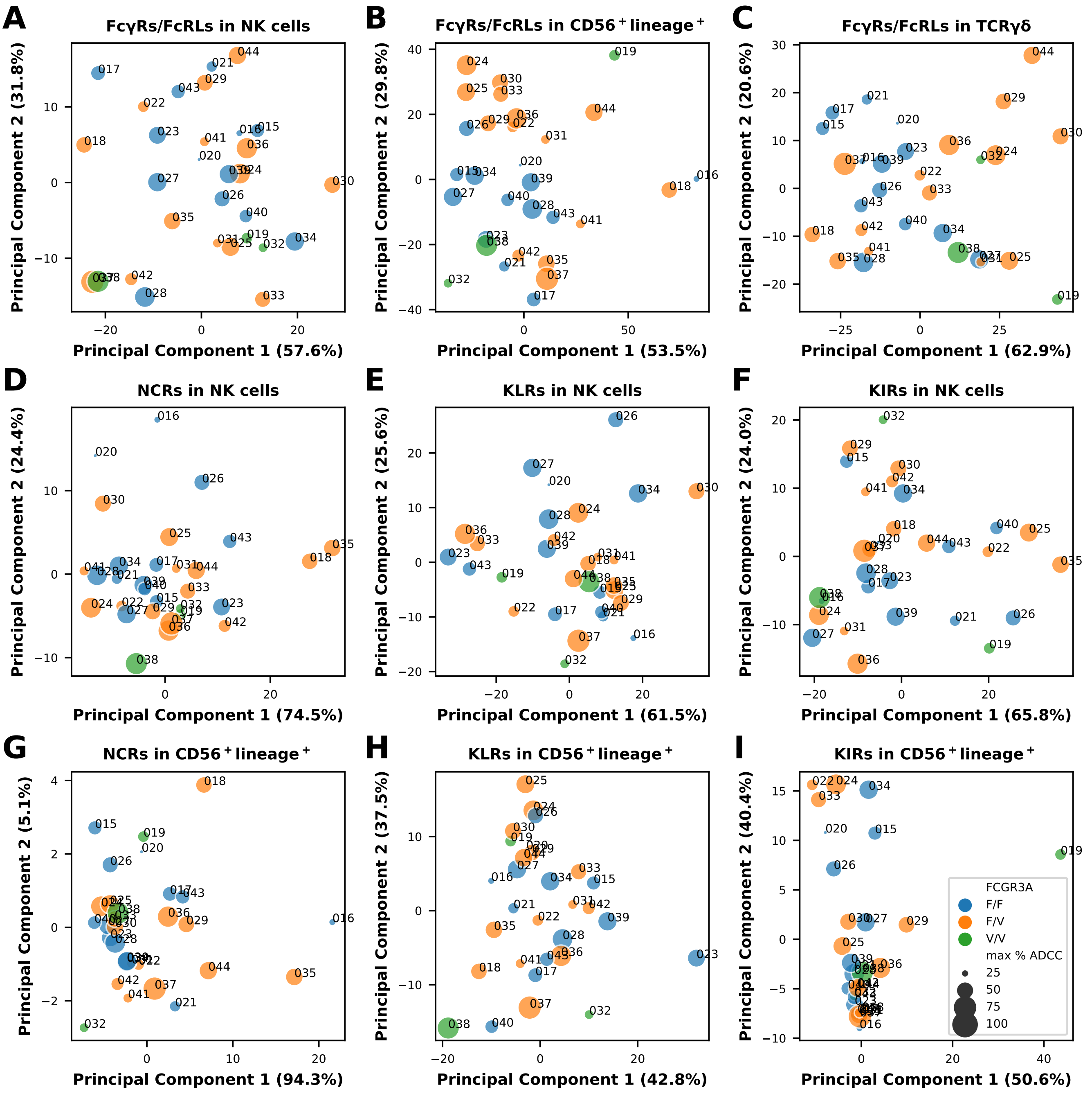

Figure S9: Complementary PCA data using a restricted number of features and focusing on specific cytotoxic cell subsets. PCA using exclusively the data of the expression of FcγRs and FcRLs in NK cells ($CD56^{+}lineage^{-}$) **(A)**, $CD56^{+}lineage^{+}$ cells **(B)** and γδ-T cells **(C)**, and the expression of the NCRs **(D,G)**, KLRs **(E,H)** and KIRs **(F,I)** in NK cells ($CD56^{+}lineage^{-}$) **(D-F)** and in total $CD56^{+}lineage^{+}$ cells **(G-I)**. Values in parentheses show the percentage variance explained by each PCA axis.

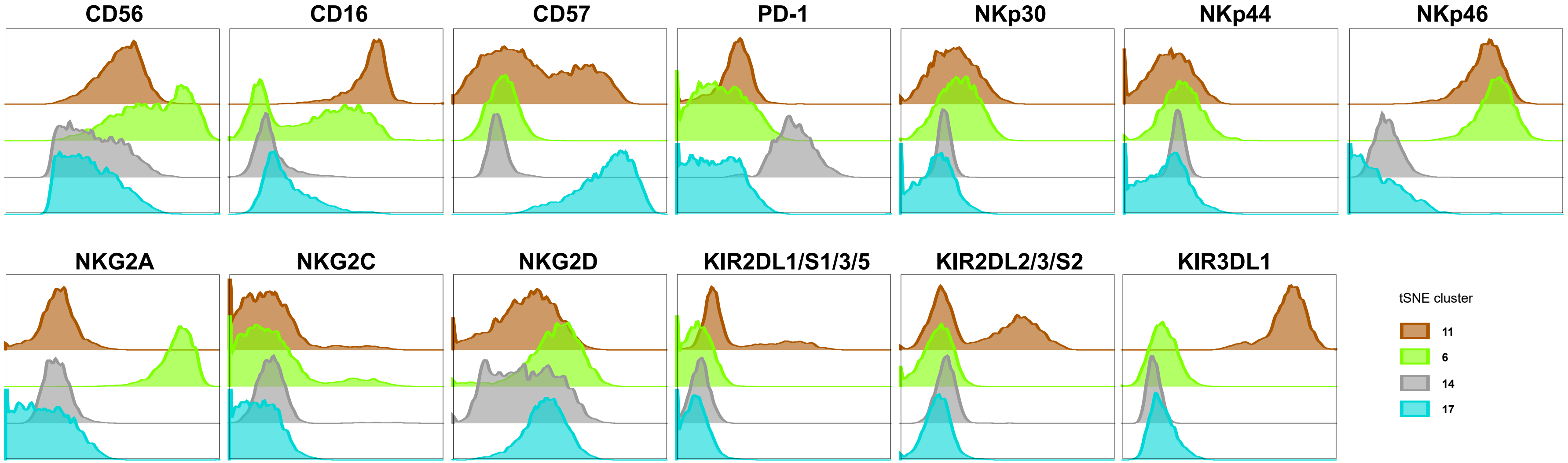

Figure S10: Histograms generated upon query gating on the dimensionality reduction analysis dot plot obtained in Figure 4B, and showing the expression of the indicated markers in the tSNE clusters *6*, *11*, *14* and *17*.

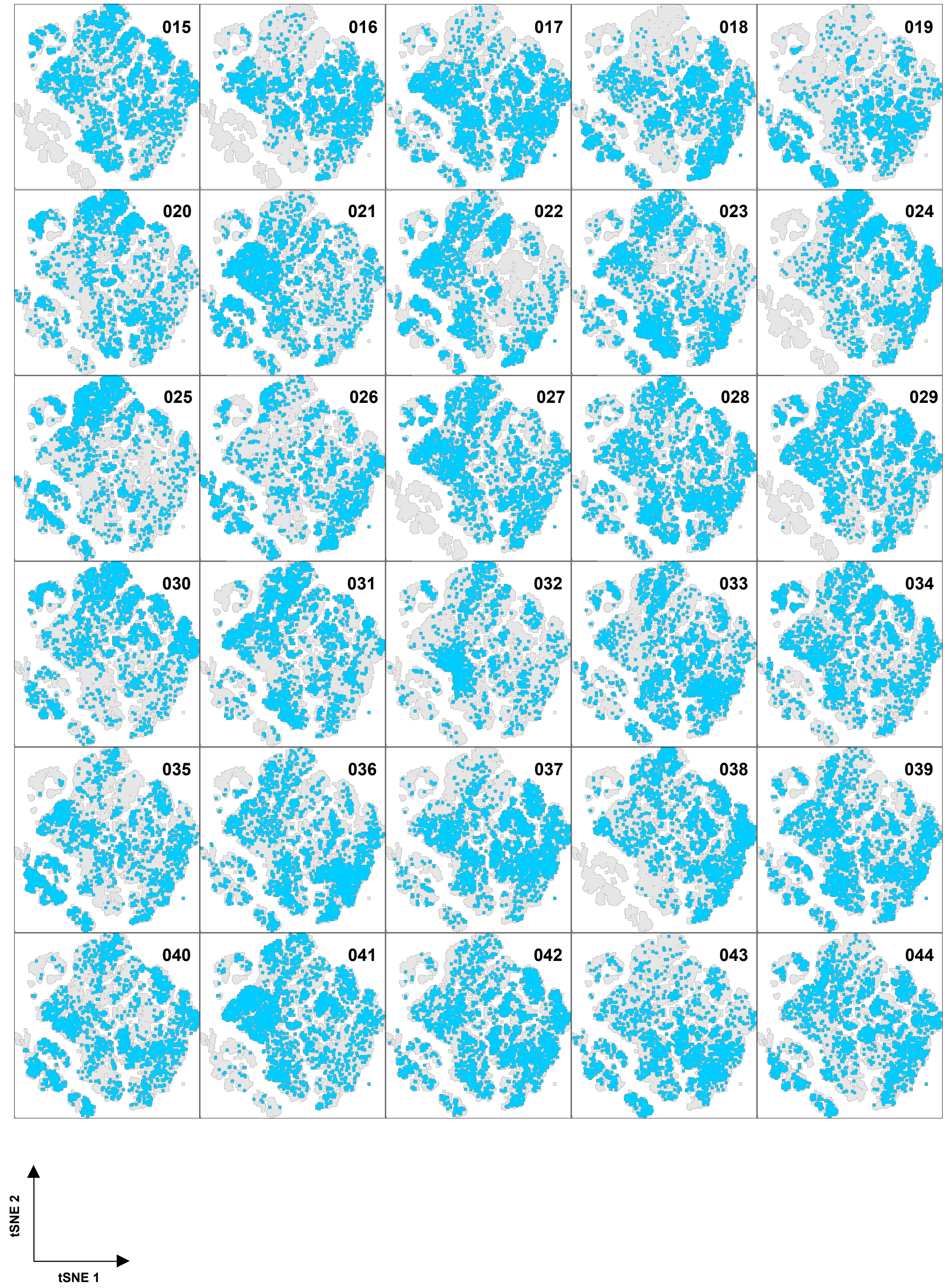

Figure S11: Projection on the dot plot resulting from the tSNE analysis (grayed out) discussed in Figure 4 showing the location of all the CD56$^+$ cells (in blue) from the indicated donor.

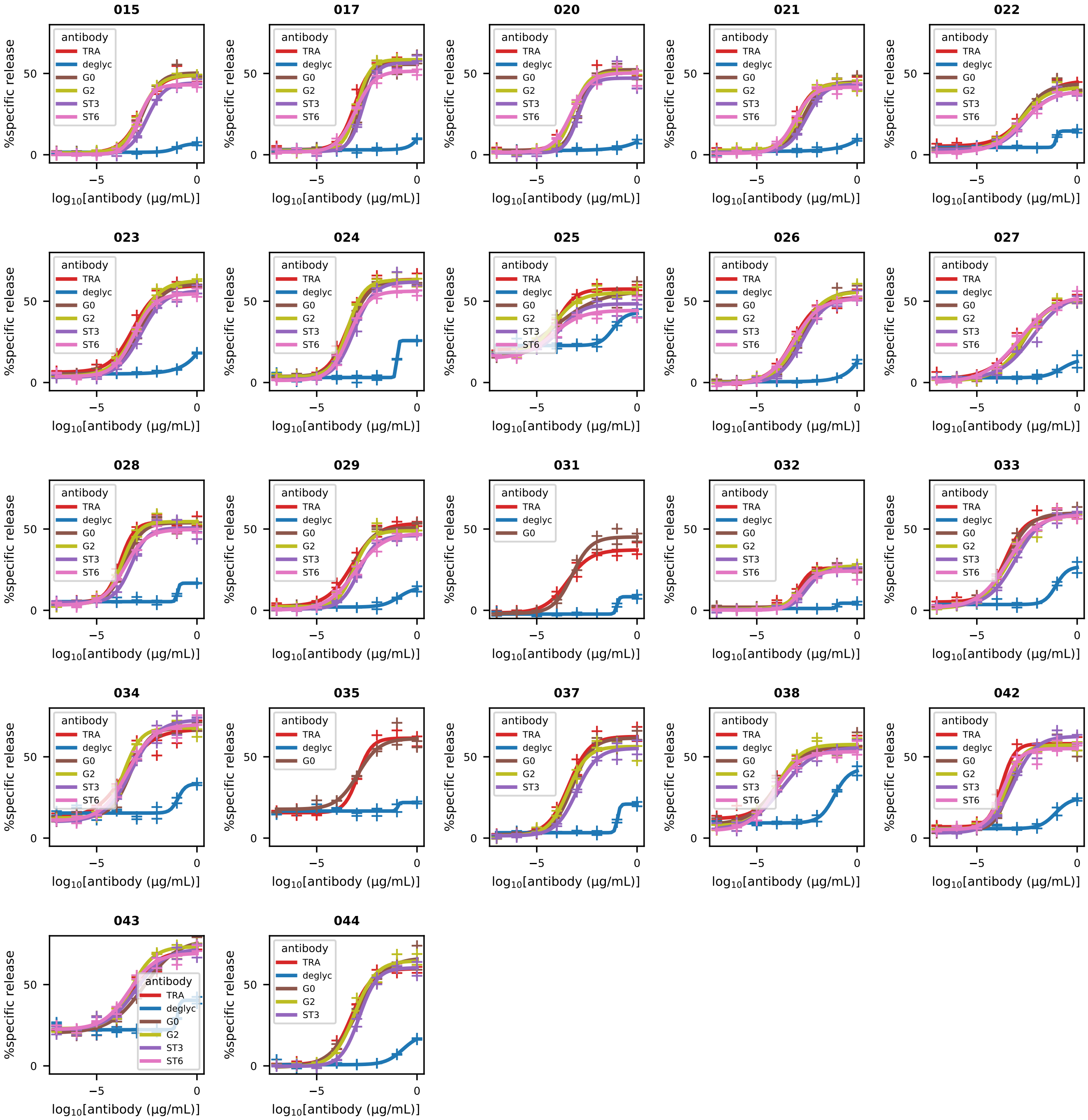

Figure S12: Additional ADCC data using isolated CD56+ as effector cells, BT474 target cells and glyco-engineered variants of trastuzumab. Raw percent of specific lysis values (in duplicate) are shown together with the curves resulting from a 4-parameter logistic model fitting for the 6 antibodies tested as indicated in the legend, at the concentrations indicated on the x-axis and for the indicated donors.
